# Observation of a phononic Mollow triplet in a multimode hybrid spin-nanomechanical system

**DOI:** 10.1038/ncomms9603

**Published:** 2015-10-19

**Authors:** B. Pigeau, S. Rohr, L. Mercier de Lépinay, A. Gloppe, V. Jacques, O. Arcizet

**Affiliations:** 1 Institut Néel, CNRS et Université Grenoble Alpes, 38042 Grenoble, France; 2 Laboratoire Charles Coulomb, Université de Montpellier and CNRS, 34095 Montpellier, France

## Abstract

Reminiscent of the bound character of a qubit's dynamics confined on the Bloch sphere, the observation of a Mollow triplet in the resonantly driven qubit fluorescence spectrum represents one of the founding signatures of quantum electrodynamics. Here we report on its observation in a hybrid spin-nanomechanical system, where a nitrogen-vacancy spin qubit is magnetically coupled to the vibrations of a silicon carbide nanowire. A resonant microwave field turns the originally parametric hybrid interaction into a resonant process, where acoustic phonons are now able to induce transitions between the dressed qubit states, leading to synchronized spin-oscillator dynamics. We further explore the vectorial character of the hybrid coupling to the bidimensional deformations of the nanowire. The demonstrated microwave assisted synchronization of the spin-oscillator dynamics opens novel perspectives for the exploration of spin-dependent forces, the key ingredient for quantum state transfer.

## Abstract (web summary)

The Mollow triplet, originally observed in the fluorescence spectrum of an optically excited two level system, is a signature of quantum electrodynamics. Here, the authors observe its phononic equivalent by magnetically coupling a single nitrogen-vacancy qubit to the vibrations of a silicon carbide nanowire.

## Graphical abstract

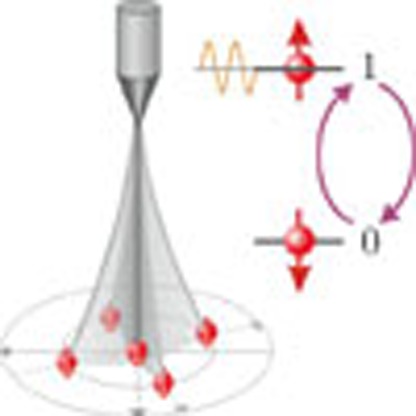

Amechanical oscillator coupled to a two-level system is a versatile basis to study the interaction between macroscopic and purely quantum objects. This unconventional combination^1^,^2^ is a promising route towards the generation of non-classical states of motion of macroscopic objects. Hybrid-coupling signatures have now been demonstrated between a mechanical oscillator and Bose–Einstein condensates^3^,^4^, superconducting qubits^5^,^6^,^7^, solid-state single spins^8^,^9^,^10^,^11^,^12^,^13^,^14^,^15^,^16^,^17^, molecules^18^ or quantum dots^19^,^20^,^21^,^22^,^23^.

The hybrid interaction coupling phonons and qubits is in profound analogy with quantum electrodynamics (QED) where hallmark experiments revealing the interplay between atoms and photons have permitted exploring the foundations of quantum mechanics. In particular, the appearance of a Mollow triplet in atomic fluorescence spectra^24^, characterized by the onset of sidebands appearing on each side of the pump frequency with splitting proportional to the laser field strength, is one of the characteristic signatures of the strongly driven Jaynes–Cumming interaction. Along with the Autler–Townes doublet^25^ or vacuum Rabi oscillations, it expresses the dressing of the atom with the optical photon field^26^. Since then, Mollow triplets were observed in atomic vapours^27^,^28^, single molecules^29^,^30^ single quantum dots^31^,^32^ or superconducting qubits^33^ coupled to photon fields in the optical or microwave (MW) domain.

Here we report on the observation of a phononic Mollow triplet, where the phonon field of a nanomechanical oscillator dresses a MW-dressed single spin-qubit immersed in a strong magnetic field gradient. We investigate the dynamics of the spin qubit in presence of large mechanical drive when the spin precession gets locked onto the mechanical-driving tone. We exploit the bidimensional deformations of the nanowire fundamental eigenmodes to fully investigate the vectorial character of the hybrid coupling. This also represent a novel dynamical regime for hybrid qubit-nanomechanical systems: the observed synchronization of the spin precession onto the mechanical oscillation frequency opens new detection strategies for observing spin-dependent forces.

## Results

### Hybrid spin-nanomechanical interaction

**Figure 1:**
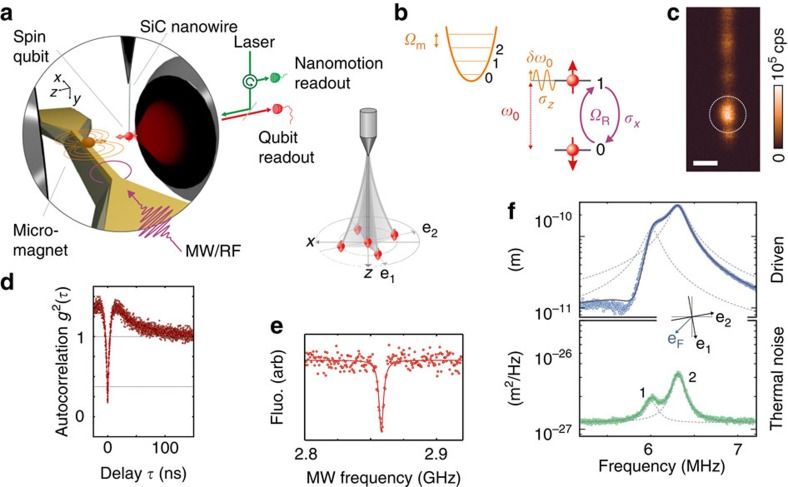
A hybrid spin-qubit-nanomechanical system. (**a**) A single NV spin-qubit hosted in a diamond nanocrystal is attached at the oscillating extremity of a suspended SiC nanowire. A strong magnetic field gradient source is micro-positioned in the vicinity of the hybrid system to magnetically couple the spin state to the vibrations of the nanoresonator through the Zeeman effect. (**b**) In the parametric coupling regime, the mechanical motion modulates the qubit energy with an amplitude *δω*_0_. *Ω*_*R*_/2*π* is the MW driven Rabi frequency (**c**) Scanning fluorescence image of the hybrid device (1 μm scale bar). (**d**) Autocorrelation function of the NV spin-qubit fluorescence revealing the presence of a single defect. (**e**) ESR spectroscopy of the suspended spin qubit in a weak magnetic field, highlighting the spin-state dependence of the average emitted fluorescence (FWHM∼4 MHz). (**f**) Brownian motion of the nanowire measured in reflection (below) revealing the two fundamental eigenmodes, which can be coherently driven through electrostatic or piezo actuations. The response curve (above) permits to determine the local orientation **e**_**F**_ of the force vector as well as its magnitude^39^.

Our hybrid device consists of a single nitrogen-vacancy (NV) spin-qubit hosted in a diamond nanocrystal attached to the vibrating extremity of a silicon carbide (SiC) nanowire^10^. A strong magnetic field gradient couples both components through a spatially dependent Zeeman effect (Fig. 1a). Formally, the generic hybrid spin-oscillator Hamiltonian can be expressed as 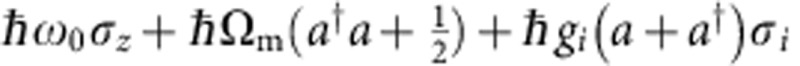, where *ω*_0_/2*π* is the qubit energy, *Ω*_*m*_/2*π* the oscillator frequency, *a*^†^(*a*) the phonon creation (annihilation) operator, *σ*_*i*_ the Pauli matrices of the spin-qubit quantized along the *z* axis and *g*_*i*_ the respective coupling constants. As in QED, a distinction between the transverse and parametric regimes can be made. In the first situation, described by the interaction Hamiltonian 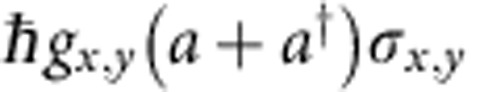, the mechanical oscillator and qubit can coherently exchange single excitations if they have similar frequencies. Several hybrid mechanical systems are exploring this regime, either through a direct interaction^6^,^34^,^35^ or mediated by a bus cavity^7^,^36^,^37^. In the case of parametric hybrid-coupling, the mechanical motion modulates the qubit energy according to the coupling Hamiltonian 
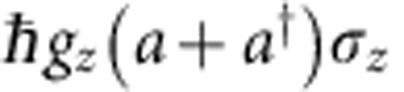
. Reciprocally, the qubit exerts a state dependent force on the oscillator which represents the key ingredient for quantum state transfer between both components. This configuration enables hybrid coupling between components with totally different excitation energies. Moreover by employing a resonant MW tone driving Rabi precession of the qubit at frequencies *Ω*_R_/2*π* close to the mechanical frequency, it is possible to let both components of the hybrid system evolve on similar time scales^15^,^38^. By doing so the parametric interaction with the original qubit is turned into a transverse coupling to the MW-dressed qubit. This configuration enables the observation of a phononic Mollow triplet, provided that the phonon field is coherently driven and that the oscillator frequency is larger than the qubit decay rate *Γ*_spin_, which corresponds to the so-called resolved sideband regime (*Ω*_*m*_>*Γ*_spin_) (ref. 16) of the parametric interaction. In that situation we introduce the dynamical parametric modulation strength *δω*_0_, which denotes the classical amplitude of the mechanically driven parametric modulation (Fig. 1b).

The bidimensional vibration properties of our nanowires were described in ref. 39. First signatures of parametric coupling of a single NV spin-qubit to the vibrations of a nanowire were reported in ref. 10 in the adiabatic regime through continuous spin-qubit electron spin resonance spectroscopy. The mechanism of spin locking on a time varying RF field was first observed in ref. 15 and suggested the possibility to observe a phononic Mollow triplet in our hybrid spin-nanomechanical system. Formally, this required entering the resolved sideband regime, developing fast and stable dynamical actuation and readout capacity of the suspended spin qubit and coping with the bidimensional character of the spin-qubit trajectories in space. This permitted thereby a full exploration of the intrinsically vectorial nature of the hybrid parametric coupling.

### Experimental setup

The experimental setup is sketched in Fig. 1a. The nanomechanical oscillator is a 6-μm-long SiC nanowire of 300 nm diameter, suspended from a sharp metallic tip. Its moving extremity is functionalized with a ≃50 nm nano-diamond hosting a single NV defect. The hybrid system is investigated with a confocal microscope apparatus (Supplementary Fig. 1) and a 532-nm pump laser. It serves for both measuring the vibrations of the nanowire using either the transmitted or reflected light beams and for optical polarization and readout of the qubit using spin-state dependent fluorescence detection^40^, see Supplementary Fig. 2.

Measurements of the nanowire Brownian motion permit determining the mechanical properties of the fundamental flexural eigenmodes^39^. These are aligned along two perpendicular directions **e**_**1,2**_, see Fig. 1f, tilted by ≈10° with respect to the optical axis, at frequencies *Ω*_*m*_/2*π* (*m*=1 or 2) of 5.99 and 6.29 MHz respectively, with mechanical damping rates 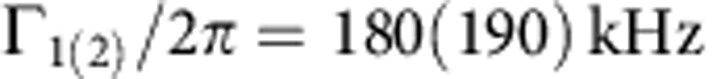 in air, limited by acoustic emission. The measured effective masses of *M*_eff_≈10^−15^ kg correspond to a spatial spreading of their Brownian motion over 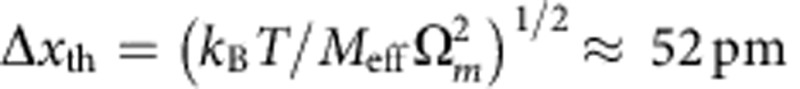 with zero-point fluctuations of Δ*x*_*q*_≈36 fm. Using a resonant force actuation *δ***F**, either piezoelectric or electrostatic, it is possible to drive vibrations of the the nanowire around its rest position **r**_**0**_. Its vectorial deflection *δ***r**(*t*) can thus be expressed in frequency space as:


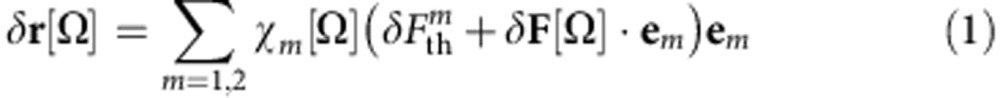


using the mechanical susceptibilities 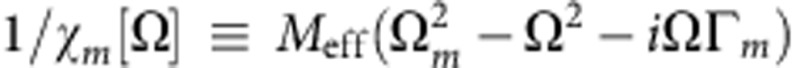 and the independent Langevin forces 
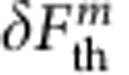
. By adjusting the drive frequency as well as the orientation **e**_**F**_ of the force vector with respect to the nanowire eigenmodes it is thus possible to generate different trajectories in the oscillation plane. This will permit exploring the vectorial character of the magnetic hybrid interaction.

The backscattered fluorescence of the NV defect is collected by avalanche photodiodes arranged in a Hanbury Brown and Twiss configuration which allows to confirm the presence of a single NV defect through fluorescence intensity autocorrelation functions (Fig. 1d; ref. 41). The qubit can be initialized in its ground state through optical pumping, manipulated with quasi-resonant MW fields and readout exploiting its spin-state dependent fluorescence rate^40^. When immersing the suspended NV defect in a strong magnetic field gradient the spin state becomes position dependent, as described by the Zeeman Hamiltonian −*gμ*_B_***σ***·**B**(**r**_**0**_+δ**r**). At first order the nanomechanical oscillator's vectorial deflection *δ***r** and the spin are dynamically coupled through a magnetic field gradient according to the interaction Hamiltonian:


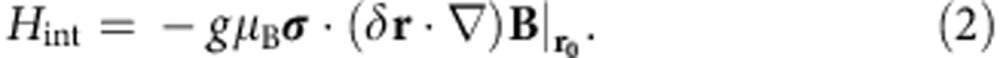


Thus, it is generally possible to tune the interaction from resonant (*σ*_*x,y*_) to parametric (*σ*_*z*_) by adjusting the qubit frequency and the topography of the magnetic field gradient. In the following, we will only investigate the parametric case, where the nanomotion modulates the qubit energy.

**Figure 2:**
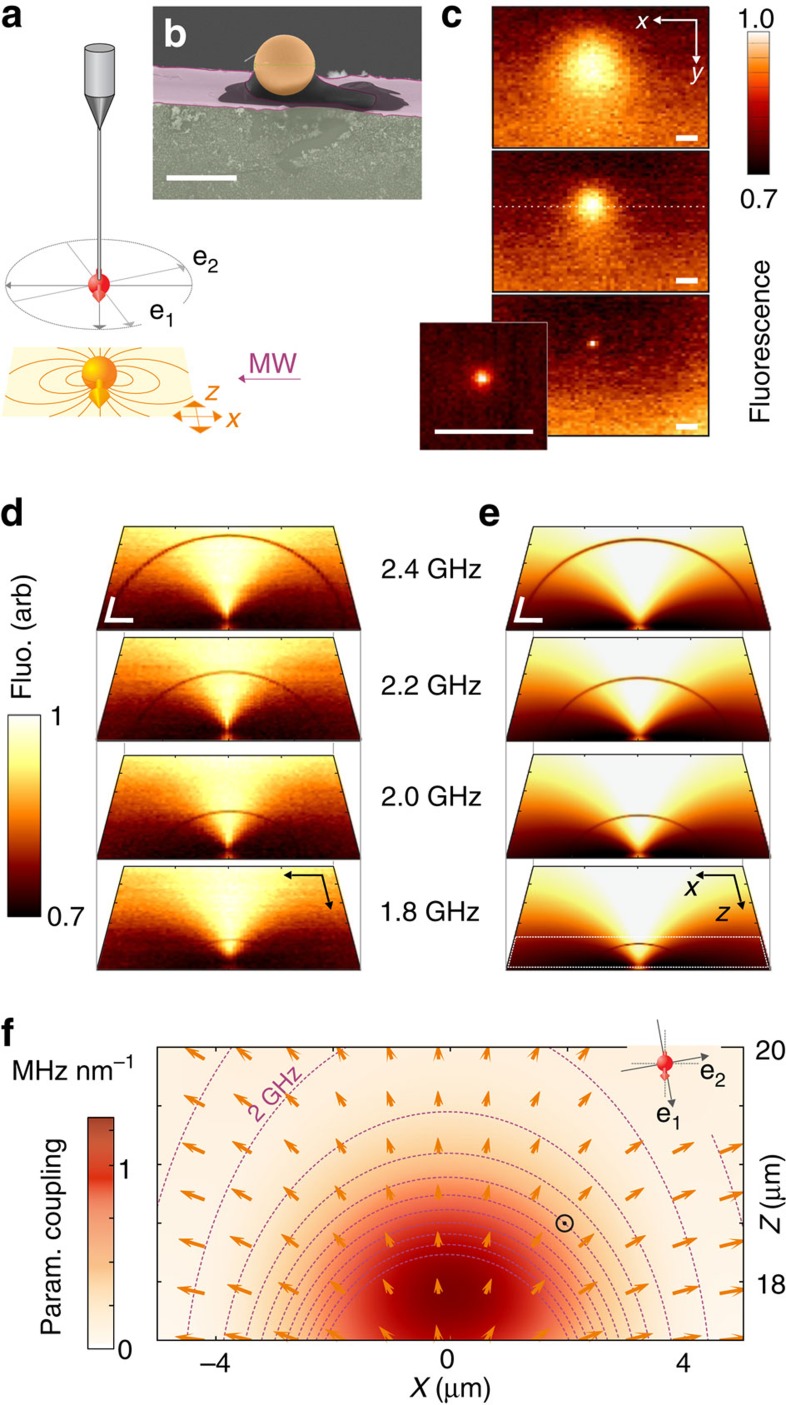
Mapping the parametric vectorial hybrid interaction. (**a**) The coupling strength is determined by measuring the spatial dependence of the spin-qubit energy 
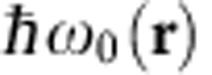
 in absence of mechanical drive for different positions of the micromagnet. (**b**) Coloured SEM image showing the 30-μm wide suspended MW waveguide supporting the NdFeB microsphere, which is piezo scanned in the vicinity of the NV (20 μm scale bar). (**c**) Fluorescence rates measured at different horizontal positions (22, 20 and 17.5 μm) while scanning the micromagnet in the vertical *x–y* plane. The mechanism of fluorescence quenching in transverse magnetic field strongly confines the suitable working points (2 μm scale bars). (**d**) Similar measurements in the horizontal *x–z* plane in presence of a single MW tone at varying frequencies (2 μm scale bars). (**e**) Corresponding numerical simulations based on a purely dipolar magnetic field (Supplementary Note 3 and Supplementary Fig. 2). The resonant slices appear as dark lines which identify the iso-spin energy surfaces 
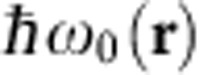
, whose spatial derivative permits determining the vectorial parametric coupling strength, 
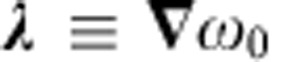
. Its projection onto the oscillation plane is shown in **f** as a color map while its orientation is indicated by the orange arrows. The dashed lines reproduce the iso-energy lines (100 MHz spacing). ⊙ indicates the working point used in the following.

A 18-μm diameter NdFeB hard magnetic sphere generates the magnetic field gradient needed to generate a large hybrid-coupling strength. It is positioned onto a narrow gold stripline antenna, see Fig. 2b, used to deliver the resonant MW field and especially designed to fit between the high numerical aperture objectives without compromising the photon collection efficiency (Supplementary Note 1) and the spatial access to the magnetic gradient source. The latter can be piezo scanned with respect to the suspended NV spin with nanometric precision. The following restrictions dictate the necessary configuration of the experimental setup. Rotating the nanowire while optimizing the collected fluorescence allows to align the intrinsic spin's quantization direction along the optical *z* axis^10^. The remanent magnetization of the NdFeB particle was oriented in a strong magnetic field (≈1.5 T) and aligned with the NV natural quantization axis to preserve the spin-selective readout efficiency. The magnetic bead stray field also allows to polarize the ^14^N nuclear spin by working at the excited state level anticrossing (**B**≈50 mT **e**_z_) (refs 42, 43), which permits to restrict our system to a pure two-level system through MW frequency selection. Finally, the last fundamental requirement consists in reaching a large parametric coupling strength, orienting **∇***B*_*z*_ with the direction of the eigenmode of interest.

### Determination of the vectorial parametric coupling strength

To determine the vectorial parametric coupling strength, the spatial dependence of the spin-qubit energy *ω*_0_(**r**) was measured by collecting the NV fluorescence while scanning the position of the micromagnet in presence of a continuous MW tone. Typical fluorescence maps are shown in Fig. 2d for varying MW frequencies. The projections of the qubit iso-energy surfaces on the oscillation plane, see Fig. 2d, appear as dark resonant slices^44^,^45^,^46^,^47^. In addition, a global fluorescence quenching which indicates regions with a strong off-axis magnetic field^48^. Reproducing this measurement for varying MW frequencies permits determination of *ω*_0_(**r**) (Fig. 2f). When moving in those strong magnetic field gradients, the suspended spin qubit undergoes a dynamical parametric energy modulation of 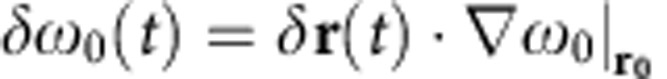, which is determined by evaluating the gradient of the iso-energy map in the (**e**_**1**_, **e**_**2**_) oscillating plane. The mapping of the vectorial coupling strength 
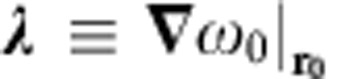
 measured in a 10 × 2.5 μm^2^ horizontal area in front of the magnetic bead is reproduced in Fig. 2f. Inherent to the dipolar structure of the microbead magnetic stray field, it strongly varies in magnitude and in orientation 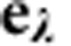 which permits a fine adjustment of the vectorial coupling strength with respect to the eigenmodes orientations by properly nano-positioning the micromagnet. Furthermore analysis of the fluorescence quenching in this imaging procedure also permits a direct identification of the locations where the **B** field is properly aligned with the NV quantization axis (Fig. 2c,d), which is a key requirement to ensure efficient optical spin-state readout. Finally, the triple requirement of avoiding fluorescence quenching, polarizing the nitrogen nuclear spin and operating with a large parametric coupling strength compete in determining the best location in space where to operate the experiment.

### Qubit dynamics in presence of coherent mechanical motion

**Figure 3:**
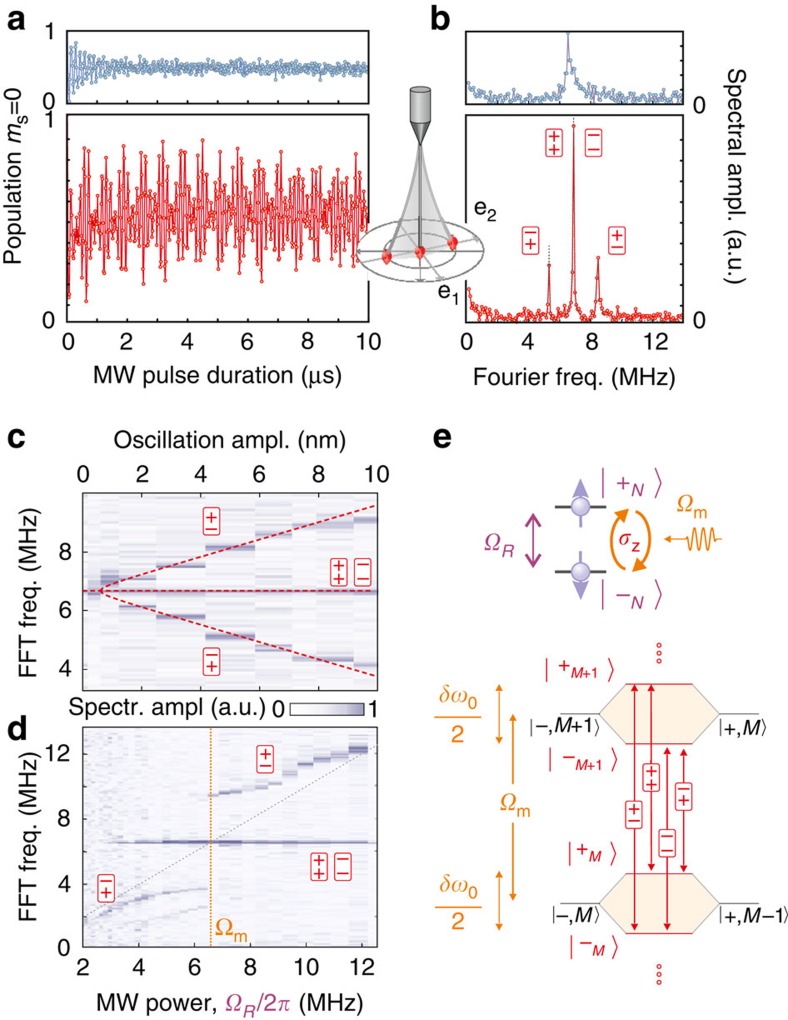
A phononic Mollow triplet. (**a**) Evolution of the spin population when the spin qubit is at rest (top) and oscillating in space (bottom) with a piezo driven 5 nm amplitude along **e**_**2**_. (**b**) Magnitude of the corresponding Fourier transforms. A characteristic triplet structure is observed when the spin is oscillating at *Ω*_d_≈*Ω*_R_. (**c**) Triplet separation as a function of the oscillation amplitude. The fit corresponds to a parametric coupling strength of 0.5 MHz nm^−1^. (**d**) Dependence of the triplet structure on the Rabi frequency *Ω*_*R*_/2*π* detuning. (**e**) The phonon field is dressing the MW-dressed qubit (see text). The measured Rabi oscillations represent a time-resolved measurement of the dipole of the dressed qubit, whose spectrum reflects the allowed transitions between the phonon-dressed multiplicities. The Mollow triplet appears when the oscillation amplitude is large enough to create a parametric energy modulation *δω*_0_ that exceeds the spin decay rate. It can be estimated to *Γ*_spin_≈100 KHz from the decay time of Rabi oscillations in the vibrating case.

Having thus fully characterized the static properties of the system, we now investigate the qubit dynamics in presence of coherent mechanical motion, generated by a modulated piezoelectric-driving force. We first restrict our analysis to one single mechanical mode (*m*=2) by positioning the gradient source at a location allowing a large parametric coupling strength along the **e**_**2**_ orientation and tuning the external drive frequency *Ω*_d_/2*π* to the resonance of the second eigenmode (6.29 MHz). The qubit is initialized in its ground state with laser illumination while the MW power is adjusted so that *Ω*_R_≈*Ω*_d_. The subsequent Rabi evolution of the population *σ*_*z*_(*t*) is shown in Fig. 3a, in absence (top) and in presence (bottom) of the coherent mechanical drive. The corresponding Fourier transforms are shown in Fig. 3b. While first a single decaying oscillation is observed, a longer-lasting beating signal can be observed when the qubit is coherently oscillating in space, presenting a characteristic triplet spectrum, whose central component coincides with *Ω*_d_, revealing the synchronization of the spin on the oscillator dynamics. Increasing the oscillation amplitude *δr*[*Ω*_d_] from 0 to 9 nm results in a linear increase of the triplet separation, see Fig. 3c, up to *δω*_0_≈2π × 5 MHz, corresponding to a 0.5 MHz nm^−1^ parametric coupling strength (20,000 T m^−1^ equivalent magnetic gradient or *g*_*z*_/2*π*=20 Hz), in good agreement with the measured coupling strength 
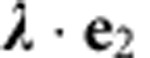
 at the target position, see Fig. 2f. Scanning the MW power permits to illustrate the dependence of the triplet structure in the detuning between *Ω*_R_ and *Ω*_d_, and underlines the synchronization of the qubit precession on the driven nanomotion^15^,^38^.

### A doubly dressed spin qubit

These observations can be explained by a double dressing of the spin qubit with MW photon and acoustic phonon fields as follows. The resonant interaction of the MW pump field with the qubit can be described in the dressed states basis 
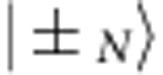
, see Fig. 3e, parameterized by the number of excitations *N* shared between the qubit and the MW pump field^49^. Under intense coherent excitation, the dynamics of the spin-MW (polariton-like) subsystem can be formally described by a pseudo qubit (
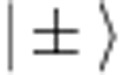
), see Supplementary Note 4, quantized along the MW polarization axis with a characteristic energy splitting of *Ω*_R_/2*π* (ref. 49). As a consequence of this rotation of perspective in the Bloch sphere, the respective roles of the *σ*_*y*_, *σ*_*z*_ operators are consequently exchanged. Therefore, the phonon field parametrically coupled to the spin qubit (∝(*a*+*a*^†^)*σ*_*z*_) is now able to resonantly drive the pseudo qubit, if the resonance condition *Ω*_R_≈*Ω*_d_ is met. This second interaction can similarly be described by a second dressing of the pseudo qubit by the phonon field. This gives rise to a ladder of phonon dressed states, see Fig. 3e, with eigenstates 
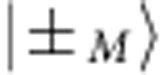
 parameterized by the number *M* of phononic and dressed qubit excitations. The energy splitting within multiplicities can be expressed as (Supplementary Note 4) 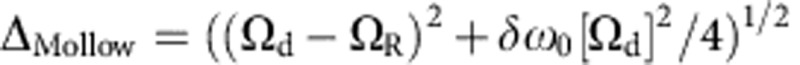, which simplifies to 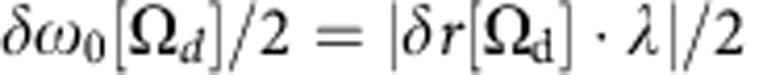 when the phononic dressing field frequency *Ω*_d_/2*π* is resonant with the dressed qubit energy splitting *Ω*_R_/2*π*. As a consequence the spectrum of Rabi oscillations is peaked at frequencies corresponding to the allowed transitions for the *σ*_*z*_ operator (Fig. 3e and Supplementary Fig. 3).

Measuring the temporal evolution of the spin-qubit population 
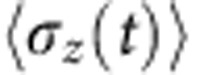
 indeed permits to record the temporal evolution of the dipole of the MW-dressed qubit (
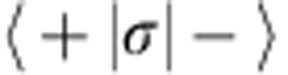
) (ref. 49), see Supplementary Note 4. This dipole governs the dressed qubit emission (in analogy with the atomic case), whose spectrum (Fig. 3b) reflects the cascade among phononic dressed states. This situation is precisely the one permitting the observation of a Mollow triplet in QED when the atomic fluorescence spectrum under intense illumination was measured^24^. An important distinction is that here the time resolved evolution of the ‘atomic' dipole (the dressed qubit) is accessible.

### Multimode phononic Mollow triplet

**Figure 4:**
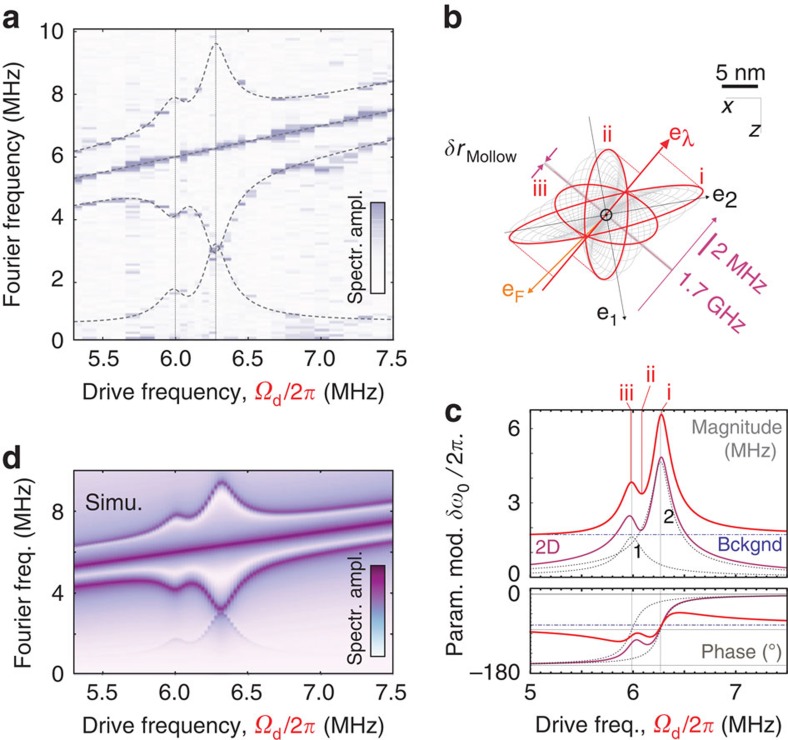
Bimodal Mollow triplet. (**a**) Mollow triplet spectra obtained while sweeping the frequency *Ω*_d_/2*π* of the electrostatic-driving force across the two mechanical eigenfrequencies, revealing the relative contributions of each eigenmodes (**e**_**1**_, **e**_**2**_) to the Mollow triplet structure. The solid lines are *δω*_0_[*Ω*_d_]/2, *Ω*_d_ and *Ω*_d_±*δω*_0_[*Ω*_d_]/2 derived from expression (3), using the determined eigenmode orientations **e**_**1,2**_, force orientation **e**_F_ and the vectorial parametric coupling strength ∇*ω*_0_ measured at the position marked by ⊙ in Fig. 2f. These quantities are reported in **b** where the spatial trajectories followed by the spin qubit are represented for varying drive frequencies. Also shown are the qubit iso-energy lines and the characteristic length *δr*_Mollow_ (see text). (**c**) The normal projections of the elliptical trajectories on the parametric coupling vector 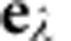 determine the Mollow triplet separation. The contribution of three specific trajectories are highlighted in panels b and c (i–iii). Also shown are the individual eigenmode contributions (dashed lines), and a 1.86 MHz residual parametric modulation (blue) caused by magnetic stray fields of the electrostatic actuation, which adds coherently to the two-dimensional phononic Mollow triplet (violet), yielding the red curve. The lower panel represents the deduced dephasing between the qubit parametric modulation and the electrostatic actuation. Both modes contribute with the same low-frequency dephasing, as expected from the respective orientations of **e**_F_, 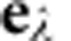 with respect to the eigenmodes at the measurement spot. (**d**) Corresponding numerical simulations (Supplementary Fig. 4) derived using the experimentally determined parameters (including a systematic 50 kHz MW detuning).

To fully explore the vectorial character of the parametric interaction, we now sweep the drive tone across both mechanical eigenfrequencies. This permits moving the qubit in both directions in the (**e**_**1**_, **e**_**2**_) oscillating plane. For each drive frequency, the MW power is adjusted to reach the resonant condition Ω_R_≈Ω_d_. The measured Mollow triplet spectra are acquired and shown in Fig. 4a. The central component of the triplets is locked onto the drive frequency *Ω*_d_, while the splitting of the Mollow triplet presents two maxima, corresponding to the response of each eigenmode. To properly understand the observed signature, it is necessary to precisely determine the spatial trajectories followed by the moving spin-qubit. To do so, an optical measurement similar to the one shown in Fig. 1c permits establishing the local orientation **e**_**F**_ and magnitude |δ***F***| of the electrostatic-driving force field^39^ and determining the driven trajectories 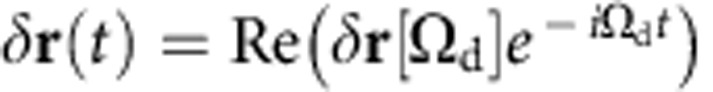 using equation (1). The slight spectral overlap between the eigenmodes leads to elliptical trajectories (Fig. 4b) which explore the oscillation plane and the magnetic field gradient over nanometric distances. Finally, the Mollow triplet's motional splitting can be adjusted with, see Supplementary Note 2:


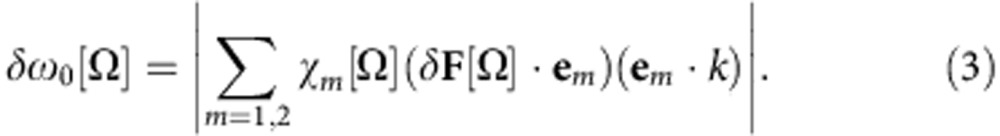


both the deduced magnitude (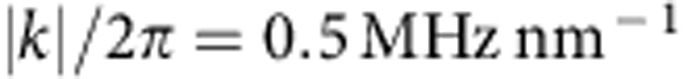) and the orientation (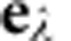 reported in Fig. 4b) of the vectorial coupling constant 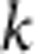 are in good agreement with the static measurements described above, at the position marked in Fig. 2f. Geometrically, the magnitude of the parametric coupling strength corresponds to the length of the projection of the ellipses on the 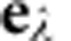 axis. Pursuing this geometrical approach, it is possible to introduce a characteristic length, 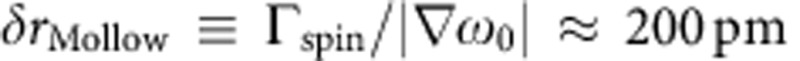, here (reported in Fig. 4b), which represents the minimum oscillation amplitude along 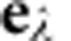 necessary to resolve the phononic Mollow triplet. It is interesting to point out that this quantity is comparable to the spatial spreading of the nanowire Brownian motion of ≈52 pm, responsible for an equivalent incoherent parametric modulation of 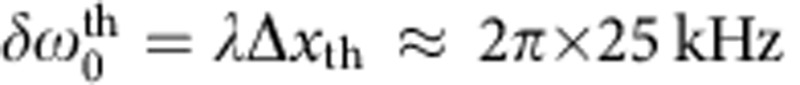, which could alter the Mollow triplet structure^50^ in larger magnetic field gradients. Understanding the coherence properties of the dressed qubit and the contribution of Brownian motion will be the subject of future investigation.

## Discussion

We have demonstrated the observation of a phononic Mollow triplet in a spin-nanomechanical hybrid system, reproducing with phonons and a spin qubit one of the founding signatures of QED based on photons and atoms. The observed signatures also demonstrate the synchronization of the spin precession onto the mechanical oscillation frequency. This opens the road towards new detection strategies for observing dynamical spin-dependent forces which can be expressed as 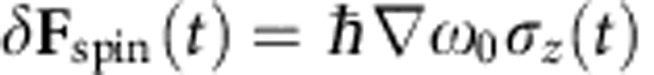. In a 10^6^Ṫm^−1^ gradient, its differential amplitude amounts to ≈20 aN, which favourably compares to the demonstrated force sensitivities falling in the aN Hz^1/2^ with our nanowires at room temperature, at the condition to make the spin-qubit precess at mechanical frequency. The required stability on the Rabi precession is however impossible to reach in a standard experimental setting but this is precisely what is enabled by the spin-locking mechanism. This opens new detection strategies for the observation of spin-dependent forces, which are the key ingredient for quantum state transfer. Also, the phononic Mollow triplet structure should also be imprinted on the oscillator dynamics through the spectrum of *σ*_*z*_[*Ω*], as mechanical sidebands, leading to a rich and novel oscillating dynamics for the oscillator. Pushing further the analogy with QED (ref. 49) where signatures of photon cascade were observed experimentally^29^, this should also provide a direct mechanism for creating single phonon sources through cascaded phonon emission within the dressed state ladder.

## Additional information

**How to cite this article:** Pigeau, B. *et al*. Observation of a phononic Mollow triplet in a multimode hybrid spin-nanomechanical system. *Nat. Commun*. 6:8603 doi: 10.1038/ncomms9603 (2015).

## Supplementary Material

Supplementary Information Supplementary Figures 1-4, Supplementary Notes 1-4 and Supplementary References
