# Supplementary material for: Observation of a phononic Mollow triplet in a multimode hybrid spin-nanomechanical system
Source: Nat Commun. 2015 Oct 19;6:8603. doi: 10.1038/ncomms9603 (PMC4634217; doi:10.1038/ncomms9603)
Supplement: Supplementary Information — Supplementary Figures 1-4, Supplementary Notes 1-4 and Supplementary References [file ncomms9603-s1.pdf]

Supplementary information for: Observation of a phononic Mollow triplet in a hybrid spin-nanomechanical system

B. Pigeau,[1] S. Rohr,[1] L. Mercier de Lépinay,[1] A. Gloppe,[1] V. Jacques,[2] and O. Arcizet[1,*]

[1] *Institut Néel, CNRS et Université Grenoble Alpes, 38042 Grenoble, France*
[2] *Laboratoire Aimé Cotton, CNRS, Université Paris-Sud and ENS Cachan, 91405 Orsay, France*

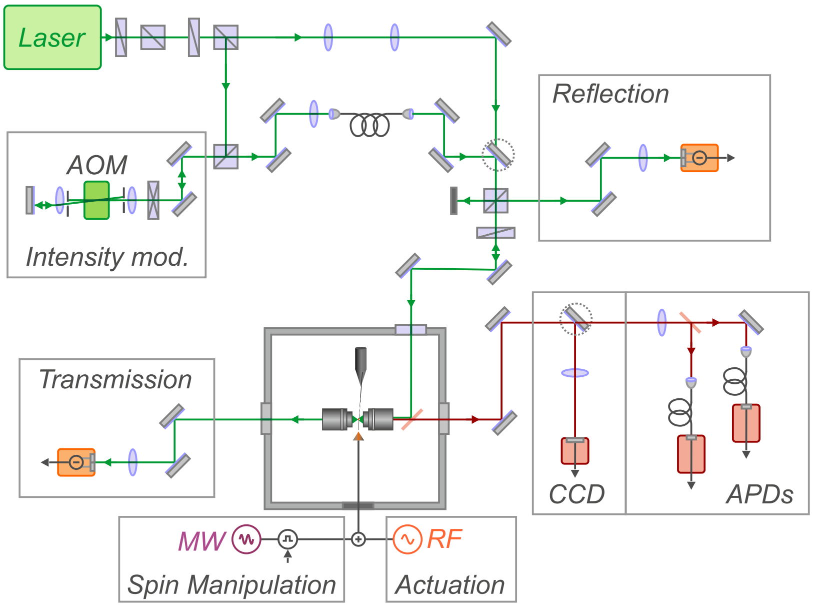

Supplementary Figure 1: Sketch of the experimental setup. AOM: acousto optic modulator, APD: avalanche photodiodes.

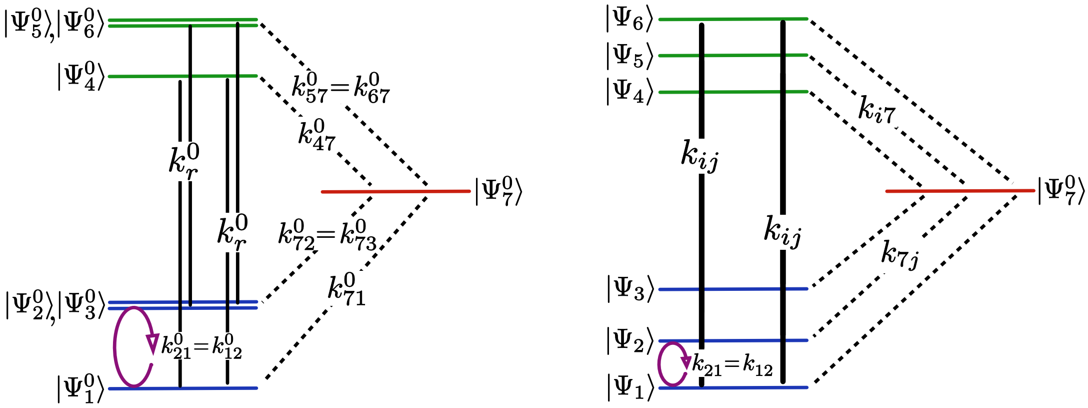

Supplementary Figure 2: Schematics of the energetic structure of the NV levels in absence (left) and presence (right) of an external magnetic field. In presence of an external magnetic field, the new eigenstates $|\Psi_i\rangle$ become mixtures of the initial eigenstates $|\Psi_i^0\rangle$. As a consequence the transition rates k_{ij} are also modified. This analysis permits to estimate the variations of the mean fluorescence rates and the evolution of the ESR contrast (see text).

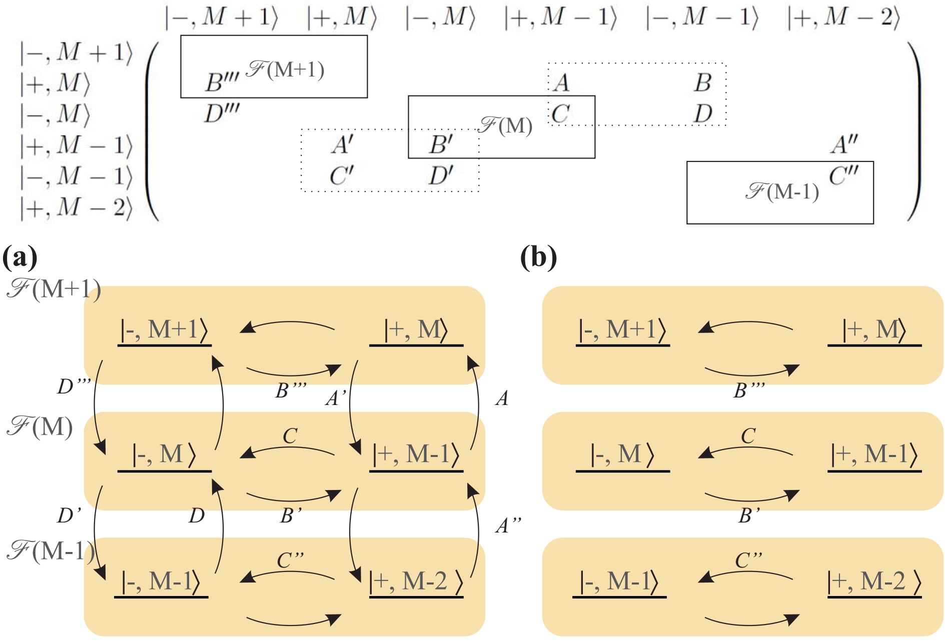

Supplementary Figure 3: Effect of the hybrid Hamiltonian on the MW dressed states $|\pm_N\rangle$. The label N for the photon number was omitted as there is no coupling between the multiplicities $\mathcal{E}_N$. The above table localizes the matrix elements of the $(\hat{a}^\dagger + \hat{a})\sigma_z$ interaction Hamiltonian. The phononic multiplicities $\mathcal{F}(M)$ are highlighted by the boxes, permitting identifying the intra-multiplicity transitions (B',C,...). The dotted frames indicate inter-multiplicity transitions. Notice that these are two fold. The C' term corresponds to a $|+_N, M\rangle \Leftrightarrow |-_N, M-1\rangle$ transition and suffers an energy penalty of $2\hbar\Omega_\mathrm{m}$. It is typically omitted by the RWA of the Jaynes-Cummings model. Contributions of such terms lead to the Bloch-Siegert corrections. A' (or D') represents the transition $|+_N, M\rangle \Leftrightarrow |+_N, M-1\rangle$, which occurs with an energy penalty $\hbar\Omega_\mathrm{m}$. They correspond to the vertical couplings shown in **(a)** and do not, as opposed to C', change the state of the singly dressed spin. The will also be averaged to zero in the second RWA. Panel **(b)** highlights the intra-multiplicity transitions, which are the only terms left after the second rotating wave approximation.

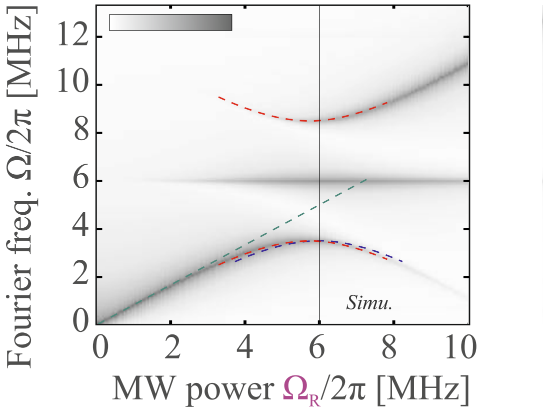

Supplementary Figure 4: Frequencies of Rabi oscillations. **(a)** The numerical simulation data are compared to the expressions in equation (64).

SUPPLEMENTARY NOTE 1: EXPERIMENTAL DETAILS

The experimental setup combines in a volume of a few microns, the NV functionalized nanowire, the magnetic field gradient source, the microwave antenna and the electrostatic actuation. An extremely high stability was required to operate the system: in a magnetic field gradient of 10^5 T/m, moving the nanowire by 1 μm shifts the static magnetic field experienced by the NV spin by 100 mT, and represents a 3 GHz frequency shift. Requiring a drift of less than 3 MHz imposes a stability of 1 nm over the measurement duration, which can last for hours. The strategy adopted was to keep the NV-functionalized nanowire fixed in space and to organize the experiment around it. We have developed an overhanging MW waveguide, supporting a magnetic microstructure providing the strong magnetic field gradient. It is also used to apply an electrostatic force to drive the spin-functionalized nanowire into motion. The optical microscope objective used for injection and for collecting the reflected pump light as well as the NV fluorescence was mounted on piezo actuators to track the NV defect in space. The whole experiment was mounted in a homemade vacuum chamber. Note that the results reported here, except for determination of the vibration orientations, were obtained at ambient pressure. However, the chamber nonetheless played a crucial role in ensuring a stable operation of the system.

Optical setup

The overall layout of the optical table is sketched in Supplementary Figure 1. The laser source employed for fluorescence measurements and optomechanical readout is a 532 nm diode pumped solid-state frequency doubled Nd:YaG laser (Laser Quantum, Torus, 200 mW maximum output power). A confocal fluorescence microscope arrangement is implemented, combined with the possibility to measure the reflected and transmitted intensities on quadrant photodiodes to read out the vibrations of the NV-functionalized nanowire. Prior to injection in the cahmber, the laser beam diameter and polarization are adjusted and its intensity modulated with an AOM in double pass followed by a fiber cleaning of the optical profile. The AOM is controlled by an arbitrary TTL pulse generator (SpinCore, PulseBlaster 500 card) in order to allow ns timing of optical pulses. The laser enters the vacuum chamber through an optical window (covered with a broadband anti-reflection coating) and is reflected by a dichroic mirror located inside the cavity. Preventing the pump beam and fluorescence channel to traverse the same optical window ensures that no additional parasitic fluorescence is detected. The laser is focused onto the functionalized nanowire and the emitted NV fluorescence is collected through a high numerical aperture microscope objective (model EC plan - NEOFLUAR 100 $\times$0.75 from Zeiss), with a 4 mm working distance. The fluorescence exiting the chamber by an optical port is then filtered to select the NV emission band, subsequently split into two paths by a polarizing beam splitter and detected by two APDs. An active electronic splitter based on logic gates duplicates the photon counts with variable delays. The different outputs are interfaced with a Nanonis scan controller, which controls the laser position, to a PCIe 6323 board (National Instruments) for ESR measurements and to a FastComTec MCS6A card for time resolved fluorescence detection, needed for the measurement of Rabi oscillations. The two APDs also permit to realize autocorrelations measurements of the emitted fluorescence. A white light source and a CCD camera (Watec 101N+) can be used for monitoring and permit a coarse alignment of the nanowire, waveguide and magnetic gradient source. The reflected intensity is collected through a 90/10 non-polarizing beam splitter which is placed on the injection path, and focused on a low noise quadrant photodiode amplifier. Similarly a second microscope objective can be installed facing the injection objective to collect the transmitted light. The DC output of both detectors can be used for fine positioning of the experiment, while the differential HF output serves for readout of the nanowire vibrations [1].

Microwave - Radiofrequency apparatus

Spin manipulation is enabled by a MW antenna which also holds the magnetic gradient source. It is brought in the vicinity of the functionalized nanowire by means of a closed loop Physik Instrument Nanocube, moving by $100\times100\times100\,\mu$m. The MW source is a SMB100A Signal Generator from Rohde & Schwarz. Its intensity is externally modulated by a fast microwave switch from Minicircuit (ZASWA-2-50DR++, coaxial High Isolation Switch) which is TTL controlled by the PulseBlaster card that governs the time sequence of dynamical spin measurements. When needed, a 25 W MW amplifier from Amplifier Research was used. Two vacuum feed-throughs with SMA connections are employed to bring MW and RF signal in and out of the vacuum chamber. An external 50Ω 2W load was placed on the output port, to avoid power dissipation inside the chamber. To implement the electrostatic actuation, two bias tees are placed upstream and downstream of the chamber MW/RF ports. When needed, a low noise RF amplifier was

used (Falco Systems), the source being either a network analyzer (Agilent 5061B) for response measurements (such as in Fig. 1f) or an arbitrary signal generator. The RF output of the second bias tee was left open in order to impose an electrostatic voltage on the nanowire and reduce RF currents at the nanowire position.

Micro-positioning and position control

As already mentioned, the functionalized nanowire serves as a spatial reference in the experiment. It is mounted on a coarse XYZ translation stage (Microcontrole, 562) equipped with piezo motors (Newport, Picomotors) that serves for coarse positioning of the nanowire on the optical axis. The injection objective is mounted on a XYZ piezostage (Physik Instrument, 733.DD) with a $30 \times 30 \times 10\,\mu$m scan range. A Nanonis scan engine serves for imaging the system and tracking the NV fluorescence in time to compensate the thermal drifts with respect to the optical axis. The piezo stage holding MW waveguide and magnetic structure is mounted on a second XYZ translation stage, that also supports the translational stage holding the nanowire. As such, after a preliminary alignment phase, where the motorized stages are involved, the nanowire ends within the scan range of the optical microscope, while the magnetic gradient source can be micro-positioned in the vicinity of the functionalized nanowire. Once this configuration is reached, all of the positioning parameters can be derived from physical measurements and optical monitoring is not needed anymore. The piezo stage holding the magnetic field gradient is controlled by a National Instrument (PCIe6323) board interfaced by a homemade scanning software.

SiC nanowires functionalized with a NV spin qubit

Long SiC nanowires with large aspect ratios and oscillation frequencies in the 100 kHz-10 MHz range exhibit large zero point fluctuations. Here we seek to demonstrate the Mollow triplet phenomena of the driven NV spin dynamics in the hybrid setup and to expose aspects of vectorial coupling in the system. Hence, we are interested in working in the spin dynamical resolved sideband regime, where the mechanical oscillation frequency surpasses the spin decay rates. Consequently, we have chosen to work with rather short nanowires.
In order to attach a single NV defect to the tip of the SiC nanowire [2], we use the arrangement of the position controllers for the nanowire and waveguide of the hybrid setup. The nanowire is mounted in a copper sample holder which is fixed to the translational stage. A drop of the nanodiamond solution (Microdiamant, 50 nm average diameter) is deposited on a Silicon wafer which is put on the same piezo stage that otherwise holds the waveguide. White light illumination of the detection volume of the confocal microscope and the CCD camera facilitate navigation of the drop of NV solution, which is steered upward until its surface is penetrated by the nanowire. We then direct green laser light onto the nanowire tip. This creates heat convection favoring a flow of nanodiamonds near the resonator. In order to limit the number of nanodiamonds attaching themselves to the wire by a surface effect, the process is interrupted after a few seconds by moving the wire out of the solution. We then use the confocal microscope and the ability to perform fluorescence scans to inspect the nanowire . If NV defects have attached to the nanowire, they become visible as bright spots in these measurements. By measuring an autocorrelation function of the light emitted from the fluorescence spots, their single photon source character can be verified. Ultimately, an ESR measurement and detection of the spin resonance near 2.87 GHz verifies that the emitters are indeed negatively charged NV defects. If no NV defect is found, the process is repeated. Despite this approach being completely heuristic, the NV yield is usually quite high and after a few iteration a single NV defect can be attached to the nanowire extremity.

Nanowire actuation

Two different actuations were used in this work: piezoelectric and electrostatic. The piezoelectric actuation was performed by driving the nanowire through a thin piezo element ($200\,\mu$m thick) glued on the nanowire support. It can be used even in absence of the RF/MW electrode, however the orientation of the corresponding force vector can neither be anticipated nor tuned. A drawback is the presence of parasitic resonances in the system response, which is not flat over the frequency range required to drive both mechanical eigenmodes (in particular in air where the damping rate is rather large). The electrostatic actuation is exerted by applying a potential between the nanowire metallic support and the waveguide, through the bias-tee inputs. The orientation of the electrostatic force vector ($\mathbf{e_F}$) can be adjusted by moving the waveguide position with respect to the nanowire. Its frequency response is extremely flat over the range of interest. This is why this strategy has been employed in the measurements illustrated in Fig. 4.

However it is responsible for a parasitic modulated magnetic stray field which partially contributes to the observations of Fig. 4. It has been taken into account in the fit of the experimental data as an additional term independent of the drive frequency $\Omega_{\rm d}$, inserted in the argument of the absolute value of equation (3), with a magnitude of 1.86 MHz and a phase of -81 degrees. This parasitic contribution is not present in case of piezo-electric actuation which does not create parasitic RF currents at the NV location.

SUPPLEMENTARY NOTE 2: FITTING OF THE MULTIMODAL AND 2D MOLLOW TRIPLET

The data of Figure 4a were fitted using the half the norm of the following expression:

$$\sum_{\rm m=1,2} \chi_{\rm m}[\Omega]\,(\delta\mathbf{F}[\Omega]\cdot\mathbf{e_m})\,(\mathbf{e_m}\cdot\boldsymbol{\lambda}) + \delta\omega_0^{\rm bckgnd} e^{i\phi_{\rm bckgnd}} \tag{1}$$

where the last complex term contains the magnetic stray field contribution visible when using the electrostatic actuation. The RF field that is employed to mechanically drive the nanowire (combined with a DC offset), is flat over the excitation frequency range, so that we can assume and verify that its phase and amplitude are frequency independent (the suspended RF-MW waveguide employed has a flat response in this frequency range). Its magnitude is $\delta\omega_0^{\rm bckgnd}/2\pi = 1.86\,\mathrm{MHz}$, with a dephasing $\phi_{\rm bckgnd}$ of $-81^{\rm o}$. The argument of the above expression can then be used to measure the dephasing between the parametric modulation undergone by the spin and the applied electrostatic voltage modulation (the electrostatic force follows instantaneously the actuation voltage).
The other fitting parameters, in particular the orientations of the force and hybrid coupling vectors are reported in Figure 4b (220° and 50° respectively). They are in agreement with the spatial map shown in Figure 2f at the measurement point, the force vector pointing towards the microbead as expected for electrostatic actuation. This orientation is also in agreement with the individual phase contributions from each of the modes, shown in Figure 4c, which are identical and equal to $-180°$ at low frequencies. In this situation, when the actuation force increases, the nanowire gets attracted towards the microbead. As a consequence both mode see the same reduction of the spin qubit energy. The situation would have been different if the $\mathbf{e_F}, \mathbf{e}_\lambda$ vectors would have lied in different quadrants with respect to the eigenmodes orientations ($\mathbf{e_1}, \mathbf{e_2}$).

SUPPLEMENTARY NOTE 3: VECTORIAL PARAMETRIC COUPLING STRENGTH

Calibration of the parametric hybrid coupling strength

This section aims at describing the static calibration of the dynamical parametric coupling strength in further detail. As outlined in the article, the ability to measure the ESR measurements at different positions in space allows to map the spatial dependance of the NV spin resonance $\omega_0(\mathbf{r})$ in the field gradient of the magnetic particle. We introduce the theoretical formalization supporting our method, and present the numerical magnetic simulations which permit modeling the spatial dependence of the vectorial parametric coupling strength used to generate Fig. 2d.

Static spatial dependence of the spin qubit energy

The static Zeeman Hamiltonian $\hat{H}_Z = -g\mu_B\hat{\sigma}_z\mathbf{B}(\mathbf{r})$ can be linearized around the chosen working point $\mathbf{r_0}$ to obtain the hybrid spin-mechanical Hamiltonian:

$$H = H_0 + H_Z^0 + H_{\rm int}, \tag{2}$$

with:

$$H_0 = \hbar\tilde{\omega}_0\sigma_z^2 \tag{3}$$

$$H_Z^0 = -g\mu_B\sigma_z\mathbf{B}(\mathbf{r_0}) \tag{4}$$

$$\hat{H}_{int} = -g\mu_B\hat{\sigma}.(\hat{\delta r}.\nabla)B|_{r_0} \tag{5}$$

Where $\tilde{\omega}_0$ is the NV spin zero field splitting (2.87 GHz). The spatial variation of the magnetic field render the spin qubit eigenstates position dependent. They are labeled $|\Psi_i(\mathbf{r})\rangle$, with $i = -1, 0, 1$ (so that in absence of magnetic field they converge towards the usual spin 1 eigenstates of the NV). Following the alignment procedure described in the main text (preserving the maximum florescence rate) permits working at a position ($\mathbf{r_0}$) where the field generated by the magnetic particle at the rest position is aligned with the NV quantization axis $\mathbf{B}(\mathbf{r_0}) = B_z.\mathbf{e_z}$ (the following calculations are however independent of exact orientation of the magnetic field at the rest position). In that case, the eigenstates of the spin qubit at the rest position are identical to the inital states.
The position dependent spin qubit energy corresponds to

$$\hbar\omega_0(\mathbf{r}) = \langle\Psi_1(\mathbf{r})|\, H_0 + H_Z\, |\Psi_1(\mathbf{r})\rangle - \langle\Psi_0(\mathbf{r})|\, H_0 + H_Z\, |\Psi_0(\mathbf{r})\rangle \tag{6}$$

which can be numerically estimated for a given magnetic source (see the following section). In practice the ordering of the energetic level structure does not vary in the region of interest, so that we can continuously follow the ESR resonance having the lowest frequency.

Vectorial parametric coupling strength

When the spin qubit moves in space, the qubit eigenstates $|\Psi_i(\mathbf{r_0} + \delta\mathbf{r})\rangle$ will vary and result in a mixture of the eigenstates obtained at the rest position $|\Psi_i(\mathbf{r_0})\rangle$. We verify here that the intuitive static calibration of the parametric coupling strength (based on the gradient of the measured $\omega_0(\mathbf{r})$ map) works as expected when one takes this intermixing into account. The parametric modulation strength $\delta\omega_0(\mathbf{r_0})$ is defined as:

$$\begin{aligned}\hbar\delta\omega_0(\mathbf{r_0}) =& \langle\Psi_1(\mathbf{r_0}+\delta\mathbf{r})|\, H_0 + H_Z^0 + H_{\text{int}}\, |\Psi_1(\mathbf{r_0}+\delta\mathbf{r})\rangle - \langle\Psi_0(\mathbf{r_0}+\delta\mathbf{r})|\, H_0 + H_Z^0 + H_{\text{int}}\, |\Psi_0(\mathbf{r_0}+\delta\mathbf{r})\rangle \\ &- \hbar\omega_0(\mathbf{r_0}).\end{aligned} \tag{7}$$

The first term can be split as $\langle\Psi_1(\mathbf{r_0}+\delta\mathbf{r})|\, H_0 + H_Z^0\, |\Psi_1(\mathbf{r_0}+\delta\mathbf{r})\rangle + \langle\Psi_1(\mathbf{r_0}+\delta\mathbf{r})|\, H_{\text{int}}\, |\Psi_1(\mathbf{r_0}+\delta\mathbf{r})\rangle$. However $|\Psi_1(\mathbf{r_0}+\delta\mathbf{r})\rangle$ is not an eigenstate of $H_0 + H_Z^0$, but at first order in $\delta\mathbf{r}$ we have:

$$\langle\Psi_1(\mathbf{r_0}+\delta\mathbf{r})|\, H_0 + H_Z^0\, |\Psi_1(\mathbf{r_0}+\delta\mathbf{r})\rangle \approx \langle\Psi_1(\mathbf{r_0})|\, H_0 + H_Z^0\, |\Psi_1(\mathbf{r_0})\rangle + \delta\mathbf{r}\cdot\mathbf{G_1} \tag{8}$$

with

$$\mathbf{G_1} \equiv \nabla\left(\langle\Psi_1(\mathbf{r_0})|\right) H_0 + H_Z^0\, |\Psi_1(\mathbf{r_0})\rangle + \langle\Psi_1(\mathbf{r_0})|\, H_0 + H_Z^0 \nabla\left(|\Psi_1(\mathbf{r_0})\rangle\right) \tag{9}$$

We have:

$$\begin{aligned}\mathbf{G_1} &= E_1(\mathbf{r_0})\left\{\nabla\left(\langle\Psi_1(\mathbf{r_0})|\right)|\Psi_1(\mathbf{r_0})\rangle + \langle\Psi_1(\mathbf{r_0})|\,\nabla\left(|\Psi_1(\mathbf{r_0})\rangle\right)\right\} \\ &= E_1(\mathbf{r_0})\nabla\left(\langle\Psi_1(\mathbf{r_0})|\,|\Psi_1(\mathbf{r_0})\rangle\right) \\ &= 0.\end{aligned} \tag{10}$$

Reproducing the same reasoning with the $|0\rangle$ state, this validates that we are only left with the interaction term:

$$\hbar\delta\omega_0(\mathbf{r_0}) = \langle\Psi_1(\mathbf{r_0}+\delta\mathbf{r})|\, H_{\text{int}}\, |\Psi_1(\mathbf{r_0}+\delta\mathbf{r})\rangle - \langle\Psi_0(\mathbf{r_0}+\delta\mathbf{r})|\, H_{\text{int}}\, |\Psi_0(\mathbf{r_0}+\delta\mathbf{r})\rangle\,. \tag{11}$$

The first term is proportional to $\langle\Psi_1(\mathbf{r_0}+\delta\mathbf{r})|\,\sigma_i\delta r_j\partial_j B_i\,|\Psi_1(\mathbf{r_0}+\delta\mathbf{r})\rangle$, which can also be written as $\delta\mathbf{r}\cdot\langle\Psi_1(\mathbf{r_0}+\delta\mathbf{r})|\,\nabla(\sigma_i B_i)\,|\Psi_1(\mathbf{r_0}+\delta\mathbf{r})\rangle$. The bracketed expression is nothing but the gradient of the total static Hamiltonian $\hat{H}_0 + \hat{H}_Z$. Since the eigenfunctions $|\Psi_1(\mathbf{r_0}+\delta\mathbf{r})\rangle$ are eigenfunctions of $H_0 + H_Z$, which does not depends upon time, the Hellmann-Feynmann theorem can be used, which states for an Hamiltonian implicitly dependant on μ (the position in our case):

$$\frac{dE_\mu}{d\mu} = \langle\Psi_\mu|\frac{dH_\mu}{d\mu}|\Psi_\mu\rangle. \tag{12}$$

Therefore one writes the parametric coupling strength as:

$$\hbar\delta\omega_0(\mathbf{r_0}) = \delta\mathbf{r}\cdot\left(\nabla E_1|_{\mathbf{r_0}} - \nabla E_0|_{\mathbf{r_0}}\right) \tag{13}$$

and we formally recognize here the spatial gradient of $\omega_0(\mathbf{r})$ (see equation (6)). As such, the parametric interaction hamiltonian can be expressed as:

$$H_{int} = \hbar\big(\delta r.\lambda\big)\sigma_z \tag{14}$$

using the vectorial coupling constant λ:

$$\lambda = \nabla\omega_0|_{\mathbf{r_0}}, \tag{15}$$

as employed in the article.

Magnetic simulations

We have performed numerical simulations of the spatial dependence of the spin energy $E(\mathbf{r_0})$ to model the energy maps obtained in figure 2d, 2e of the article. The NV fluorescence was experimentally recorded as a function of the spin position in the magnetic field gradient in presence of a fixed microwave tone. To be able to reproduce both the position of resonance slices and the spatial dependance of the fluorescence, a detailed model of the NV center must be considered. Following [3], the fluorescence properties are fully understood using a seven level model of the NV center, see Supplementary Figure 2: a spin triplet for both ground and excited states and a dark metastable singlet state labeled $|\Psi^0_{1\to7}\rangle$.

The spin ground states $|m_S = -1,0,1\rangle = |\Psi^0_{1,2,3}\rangle$ and excited states $|m_S = -1,0,1\rangle = |\Psi^0_{4,5,6}\rangle$ are respectively eigenstates of the Hamiltonian $H_{gs} = \hbar\tilde{\omega}_0^{gs}\sigma_z^2 - g\mu_B\boldsymbol{\sigma}.\mathbf{B}(\mathbf{r_0})$ and $H_{es} = \hbar\tilde{\omega}_0^{es}\sigma_z^2 - g\mu_B\boldsymbol{\sigma}.\mathbf{B}(\mathbf{r_0})$. If the magnetic field $\mathbf{B}(\mathbf{r_0}) = B_z(\mathbf{r_0}).\mathbf{e_z}$ is aligned along the quantization axis $\mathbf{e_z}$, the energetic structure of the spin is preserved, as well as the transition rates between eigenstates. They are defined by $k^0_{ij}(\mathbf{r_0})$.

The NdFeB particle creating the magnetic field gradients is an homogeneous sphere. Its magnetic stray field is therefore exactly modeled as a dipole of magnetic moment $\mathcal{M} = (MV).\mathbf{e_z}$, where M is the zero field magnetization and V the volume of the particle:

$$\mathbf{B}(\mathbf{r_0}) = \frac{\mu_0}{4\pi}\left(\frac{3(\mathcal{M}.\mathbf{r_0})\mathbf{r_0}}{r_0^5} - \frac{\mathcal{M}}{r^3}\right) \tag{16}$$

In the general case when a transverse magnetic field is present the previous Hamiltonians are no longer diagonal which modify the eigenstates as follow:

$$|\Psi_i\rangle = \sum_{j=1}^{7}\alpha_{ij}(\mathbf{r_0})|\Psi^0_j\rangle \tag{17}$$

The coefficients $\alpha_{ij}(\mathbf{r_0})$ and the new eigenfrequencies $\omega_{i\to j}(\mathbf{r_0})$ are numerically computed with the assumption that $|\Psi_7\rangle = |\Psi^0_7\rangle$. As a consequence the transition rates between the new eigenstates are given by:

$$k_{ij}(\mathbf{r_0}) = \sum_{p=1}^{7}\sum_{q=1}^{7}|\alpha_{ip}(\mathbf{r_0})|^2|\alpha_{jq}(\mathbf{r_0})|^2 k^0_{pq}(\mathbf{r_0}) \tag{18}$$

The field dependent NV fluorescence rate can therefore be calculated at each position:

$$F(\mathbf{r_0}) = \sum_{i=4}^{6}\sum_{j=1}^{3}\bar{n_i}k_{ij}(\mathbf{r_0}) \tag{19}$$

where the averaged population of each states $\bar{n_i}$ is retrieved by solving the rate equation of the system in the steady state:

$$\frac{dn_i}{dt} = \sum_{j=1}^{7}(k_{ji}n_j - k_{ij}n_i) = 0 \tag{20}$$

The microwave field with a fixed frequency ω_{MW} is only able to drive the $|\Psi_1\rangle \to |\Psi_2\rangle$ transition at the position where the resonant condition $\omega_0(\mathbf{r_0}) \equiv (E_1(\mathbf{r_0}) - E_1(\mathbf{r_0})/\hbar = \omega_{MW}$ is met. At this position the fluorescence rate is reduced by the ESR contrast:

$$C(\mathbf{r_0}) = \frac{F(\mathbf{r_0}, k_{12} = 0) - F(\mathbf{r_0}, k_{12})}{F(\mathbf{r_0}, k12 = 0)} \tag{21}$$

At each spatial position of the NV center in the magnetic field $\mathbf{B}(\mathbf{r_0})$ the optically detected magnetic resonance signal is modeled with a Lorentzian curve $L(\omega, \mathbf{r_0})$ of amplitude $F(\mathbf{r_0}).C(\mathbf{r_0})$ and linewidth Γ_S centered at $\omega = \omega_0(\mathbf{r_0})$. The resulting fluorescence rate at position $\mathbf{r_0}$ is given by $F(\mathbf{r_0}) - L(\omega_{MW}, \mathbf{r_0})$, which allows to reconstruct the fluorescence map of figure 2e.

The typical zero field transition rates used in this simulation, extracted from the literature [3], are given in the following table:

k_r	k_{12}	k_{47}	$k_{57} = k_{67}$	$k_{72} = k_{73}$	k_{71}
67 μs^{-1}	10 μs^{-1}	6.4 μs^{-1}	50 μs^{-1}	0.6 μs^{-1}	0.8 μs^{-1}

(22)

This simulation is able to reproduce and understand all the experimental features which appear in Figure 2d and validates the static estimation of the parametric coupling strength calibration procedure presented in the article. The distance to the bead being fixed, the only free parameter being the magnetic moment of the NdFeB particle, which is evaluated to be $\mathcal{M} \simeq 1.5\,10^{-9}$ A.m^2. This value is compatible with the magnetization specified for this material ($M = 5.5\ 10^6$ A.m^{-1}) which gives for a radius of $R = 9$ μm: $\mathcal{M} = M\frac{4}{3}\pi R^3 = 1.7\ 10^{-8}$ A.m^2.

SUPPLEMENTARY NOTE 4 : DYNAMICS OF THE QUBIT AND THE DRESSED QUBIT

Bloch equations

The goal of this section is to establish a set of Bloch equations that we will use to describe the evolution of the coupled spin-oscillator system.

We will consider the magnetic field coupling of a spin 1 system to a single mechanical mode in a most generic approach. Assuming that the mechanical oscillator is classical, the 2D vibrations of its oscillating extremity are denoted by $\delta\mathbf{r}$. We consider the quantum master equation for the density matrix ρ:

$$i\hbar\dot{\rho} = [H, \rho] + i\hbar\dot{\rho}_{\text{relax}}, \tag{23}$$

where the Hamiltonian H is written as

$$H = \hbar\omega_0\sigma_z^2 - g\mu_B\boldsymbol{\sigma}\cdot(\mathbf{B_0} + \mathbf{B}_{\text{MW}}(t) + (\delta\mathbf{r}\cdot\boldsymbol{\nabla})\mathbf{B}), \tag{24}$$

which includes the coupling term

$$\boldsymbol{\mu}(\delta\mathbf{r})\cdot\mathbf{e_i} = \delta r_j\partial_j B_i. \tag{25}$$

The evolution of $\boldsymbol{\sigma}$ can be written:

$$\frac{d\boldsymbol{\sigma}}{dt} = \Big(\omega_0\mathbf{e_z} + \frac{g\mu_B}{\hbar}\left(\mathbf{B_0} + \mathbf{B}_{\text{MW}}(t) + (\delta\mathbf{r}\cdot\boldsymbol{\nabla})\,\mathbf{B}\right)\Big)\wedge\boldsymbol{\sigma} + \dot{\boldsymbol{\sigma}}_{\text{relax}} + \dot{\boldsymbol{\sigma}}_{-1}, \tag{26}$$

where we have separated the contribution of the coherences originating from the $|-1\rangle$ state: $\dot{\boldsymbol{\sigma}}_{-1} = -\sqrt{2}\omega_0\begin{pmatrix} i\,(\rho_{-10} - \rho_{0\,-1}) \\ \rho_{-10} + \rho_{0\,-1} \\ 0 \end{pmatrix}$. The last step allows us to restrict our analysis to the $\{|0, |1\rangle\rangle\}$ subspace for the evolution of the spin qubit instead of a spin 1 three level system. Such a simplification permits a straightforward interpretation of the evolution of the spin populations and coherences on the Bloch sphere. This restriction to the $\{|0, |1\rangle\rangle\}$ subspace is experimentally justified in the conditions described in the main text.

The Hamiltonian can then be reduced to:

$$H/\hbar = (\omega_0 + \delta\omega_0)\,|1\rangle\langle 1| + (\Omega_{\text{R}}(t) + \delta\omega_{\text{R}})(|0\rangle\langle 1| + |1\rangle\langle 0|) \tag{27}$$

while the damping contribution is

$$\dot{\rho}_{\rm relax} = \begin{pmatrix} -\Gamma_{\rm p}\rho_{11} - \frac{1}{2}\Gamma_1(\rho_{11}-\rho_{00}) & -\Gamma_2\rho_{01} \\ -\Gamma_2\rho_{10} & +\Gamma_{\rm p}\rho_{11} + \frac{1}{2}\Gamma_1(\rho_{11}-\rho_{00}) \end{pmatrix}, \tag{28}$$

where Γ_p conveys the role of the light induced polarization of the spin qubit through optical pumping [4]. The time evolution of the density matrix elements $\rho_{ij} = \langle i|\,\rho\,|j\rangle$, given by $\dot{\rho} = \frac{1}{i\hbar}[H_{\rm tot},\rho] + \dot{\rho}_{\rm relax}$ can be expressed as:

$$\dot{\rho}_{11} = \; -i(\Omega_{\rm R}(t) + \delta\omega_{\rm R}(t))(\rho_{01}-\rho_{10}) - \Gamma_{\rm p}\rho_{11} - \frac{1}{2}\Gamma_1(\rho_{11}-\rho_{00}) \tag{29}$$

$$\dot{\rho}_{10} = \; -i(\omega_0 + \delta\omega_0(t))\rho_{10} - i(\Omega_{\rm R}(t) + \delta\omega_{\rm R}(t))(\rho_{00}-\rho_{11}) - \Gamma_2\rho_{10} \tag{30}$$

$$\dot{\rho}_{01} = \; i(\omega_0 + \delta\omega_0(t))\rho_{01} + i(\Omega_{\rm R}(t) + \delta\omega_{\rm R}(t))(\rho_{00}-\rho_{11}) - \Gamma_2\rho_{01} \quad . \tag{31}$$

We have used the time dependence of the resonant MW field $\Omega_{\rm R}(t) = \Omega_{\rm R}\cos\omega t$ (it is assumed to be aligned along the x direction, but the physics does not change in case of a y orientation). We have introduced the parametric modulation strength $\delta\omega_0(t)$ as well as the resonant contribution $\delta\omega_{\rm R}(t)$ due to mechanical oscillation in the magnetic field gradient which both vary at the timescale of the oscillator. By going into the MW rotating frame, we define:

$$\rho_{10} \equiv \tilde{\rho}_{10}e^{-i\omega t}, \quad \rho_{01} \equiv \tilde{\rho}_{01}e^{i\omega t}. \tag{32}$$

We introduce the coordinates of the Bloch vector:

$$u \equiv {\rm Re}\left(\tilde{\rho}_{01}\right), \qquad v \equiv {\rm Im}\left(\tilde{\rho}_{01}\right), \qquad w \equiv \frac{1}{2}(\rho_{11}-\rho_{00}), \tag{33}$$

and obtain the Bloch equations:

$$\begin{pmatrix} \dot{u} \\ \dot{v} \\ \dot{w} \end{pmatrix} = \begin{pmatrix} -\Gamma_2 & \delta - \delta\omega_0(t) & -2\delta\omega_{\rm R}(t)\sin\omega t \\ -\delta + \delta\omega_0(t) & -\Gamma_2 & -\Omega_{\rm R} - 2\delta\omega_{\rm R}\cos\omega t \\ 2\delta\omega_{\rm R}\sin\omega t & \Omega_{\rm R} + 2\delta\omega_{\rm R}(t)\cos\omega t & -\Gamma_1 - \Gamma_{\rm p} \end{pmatrix} \begin{pmatrix} u \\ v \\ w \end{pmatrix} - \begin{pmatrix} 0 \\ 0 \\ \Gamma_{\rm p}/2 \end{pmatrix}. \tag{34}$$

Numerical simulations

The previous set of equations are used to numerically simulate the dynamics of the spin qubit in presence of mechanical motion which generates parametric energy modulation. The time evolution of the population $\sigma_z(t)$ can then be simulated, and its Fourier transform is then calculated. The presence of light can be turned on (for ESR measurements) or off (for the Rabi dynamics) by turning Γ_p on or off.

To realize the numerical simulations displayed in figure 4d of the manuscript we proceed as follow. The parametric modulation strength $\delta\omega_0[\Omega_{\rm d}]$ is deduced for each drive frequency $\Omega_{\rm d}$ as explained in the manuscript by analyzing the driven trajectories in the parametric coupling landscape. At t=0 the spin qubit is supposed to be optically initialized in its ground state, $(u = 0, v = 0, w = -1/2)$, the mechanically induced dynamical change of the MW-spin qubit interaction strength is neglected here ($\delta\omega_{\rm R}$=0), the light is turned off ($\Gamma_{\rm p} = 0$) and we emloy a time modulated parametric modulation strength: $\delta\omega_0(t) = \delta\omega_0\sin(\Omega_{\rm d}t + \phi)$, ϕ being an arbitrary dephasing parameterizing the state of the parametric modulation at the beginning of the Rabi measurement protocol. Then the 3 coupled Bloch equations are solved with a Runge-Kutta algorithm of fourth order and the evolution of $\sigma_z(t)$ is recorded as a function of time. A sufficiently small time step is employed (100 points per oscillation period) and the duration of the simulation is chosen to ensure a proper sampling of the patterns appearing in the subsequent Fourier Transform which is realized on the temporal traces. In order to account for the absence of synchronization of our measurement protocol with the driving force, we realize numerical simulations of $\sigma_z(t)$ for varying time delays (4 different values of ϕ are used, sampling 2π) and average the obtained results. This procedure has been used to simulate Figure 4d and Supplementary Figure 4.

In our experiment the intra-multiplicity transition at $\delta\omega_0[\Omega_{\rm d}]/2$ can be seen, traducing the presence of a slight detuning between the MW field and the spin qubit resonance, which can change due to thermal drifts. Each measurement of Rabi oscillations is typically accumulated for one hour and paused every 5 minutes approximately to realize an ESR

measurement. This permits to adjust the microwave frequency to the qubit resonance, which slightly shifts due to spatial drifts in the strong magnetic gradient. A 50 kHz detuning is an average value of the qubit resonance shit measured between two relocking steps of the microwave frequency onto the qubit resonance. It is a good estimation of the average detuning one can expect in our measurements. It has thus been taken into account in our numerical simulations.

Interpretation of the Mollow triplet in terms of a double dressing

We formalize here the two-tone interaction (MW and phonon fields) with the NV spin qubit in terms of a double dressing of the spin. We begin by considering the effect of the quasi-resonant MW field on the spin enabling a first dressing. Then, we investigate the role of the mechanical phonon field which is resonantly coupled to the MW dressed qubit. We explain the triple peak features and anti-crossing in the FFT spectra.
We focus here on the case of a single mechanical mode and investigate its contribution to the phononic Mollow triplet. The generalization to two eigenmodes, driven out of resonance is straightforward and detailed in the manuscript.
The total Hamiltonian is

$$H_{\rm tot} = H_0 + H_{\rm MW} + H_{\rm int} \tag{35}$$

with

$$H_0 = \hbar\omega_0\hat{\sigma}_z + \hbar\Omega_{\rm m}\hat{a}^\dagger\hat{a} + \hbar\omega\hat{b}^\dagger\hat{b}, \tag{36}$$

$$H_{\rm MW} = \frac{\hbar\Omega_{\rm R}^v}{2}(\hat{b}^\dagger + \hat{b})(\sigma^+ + \sigma^-), \tag{37}$$

$$H_{\rm int} = \hbar g_z(\hat{a}^\dagger + \hat{a})\sigma_z, \tag{38}$$

where we have included the interaction of the microwave and phonon fields (described by the $\hat{a}, \hat{a}^\dagger$ and $\hat{b}, \hat{b}^\dagger$ annihilation and creation operators respectively) on the spin qubit, using the matrices

$$\sigma_x = \begin{pmatrix} 0 & 1 \\ 1 & 0 \end{pmatrix}, \sigma_y = \begin{pmatrix} 0 & -i \\ i & 0 \end{pmatrix}, \sigma_z = \begin{pmatrix} 1 & 0 \\ 0 & 0 \end{pmatrix}. \tag{39}$$

$\omega/2\pi$ is the MW frequency. The generic eigenstates of H_0 are $|m_S, N, M\rangle$, where $m_S = 0, 1$ is the spin state, N the photon number and M the phonon number. Their eigenenergies $E_{|m_s,N,M\rangle}$ are (setting the ground state ($|0,0,0\rangle$) energy $(\hbar\omega + M\hbar\Omega_{\rm m})/2$ to 0):

$$E_{|m_s,N,M\rangle} = m_s\hbar\omega_0 + N\hbar\omega + \hbar\Omega_{\rm m}. \tag{40}$$

Dressing the spin qubit with the MW field

We first consider the action of $H_{\rm MW}$, which does not affect the phonon field. We use the rotating wave approximation, which corresponds to dropping the off-resonant terms($\hat{b}^\dagger\sigma^+$ and $\hat{b}\sigma^-$) in $H_{\rm MW}$. This is justified for intense coherent MW fields with mean phonon numbers $\bar{N}$ as long as $\Omega_{\rm R}^v\sqrt{N} \equiv \Omega_{\rm R} \ll \omega_0$. We obtain

$$H_{\rm MW}^{\rm RWA} = \frac{\hbar\Omega_{\rm R}^v}{2}(\hat{b}^\dagger\sigma^- + \hat{b}\sigma^+). \tag{41}$$

The coupling term of the so called *Jaynes-Cummings model* [5] corresponds to interactions where a spin excitation is traded for a MW field excitation. It only couples between sets of states $\{|0,N\rangle, |1,N-1\rangle\}$. We therefore introduce the multiplicities $\mathcal{E}(0) \equiv \{|0,0\rangle\}$ and for $N \geq 1$

$$\mathcal{E}(N) \equiv \{|1,N-1\rangle, |0,N\rangle\}. \tag{42}$$

The Hamiltonian describing the spin-MW subsystem in the RWA approximation does not couple different multiplicities and can then be written as an infinite tensor product

$$H_{\text{tot}} + H_{\text{MW}}^{\text{RWA}} = \bigotimes_{N=0..\infty} H^{(N)}, \tag{43}$$

with $H^{(0)} \equiv 0$. Introducing the MW detuning $\delta \equiv \omega - \omega_0$, the Hamiltonian $H^{(N)}$ acting in the $\mathcal{E}(N)$ subspace reads

$$H^{(N)} = \hbar \begin{pmatrix} N\omega - \delta & \frac{\Omega_{\text{R}}^{v}\sqrt{N}}{2} \\ \frac{\Omega_{\text{R}}^{v}\sqrt{N}}{2} & N\omega \end{pmatrix}. \tag{44}$$

Diagonalization of the Hamiltonian leads to new eigenstates $|\pm_N\rangle$ with energies

$$E_{|\pm_N\rangle}/\hbar = N\omega - \frac{\delta}{2} \pm \frac{1}{2}\sqrt{\delta^2 + N{\Omega_{\text{R}}^{v}}^2} \tag{45}$$

featuring a splitting $\hbar\Delta_N$ with

$$\Delta_N \equiv \sqrt{\delta^2 + N{\Omega_{\text{R}}^{v}}^2}. \tag{46}$$

Using

$$\tan 2\theta_N \equiv -\frac{\Omega_{\text{R}}^{v}\sqrt{N}}{\delta} \quad \text{with} \quad \theta_N \in [0, \pi/2] \tag{47}$$

and

$$\cos\theta_N = \sqrt{\frac{1 - \delta/\Delta_N}{2}}, \ \sin\theta_N = \sqrt{\frac{1 + \delta/\Delta_N}{2}}, \tag{48}$$

the new eigenstates can be written:

$$\begin{aligned} |+_N\rangle &= \cos\theta_N \, |1, N-1\rangle + \sin\theta_N \, |0, N\rangle \,, \\ |-_N\rangle &= -\sin\theta_N \, |1, N-1\rangle + \cos\theta_N \, |0, N\rangle \end{aligned} \tag{49}$$

They are called the *dressed* states. Importantly, the interaction between MW and spin causes an energy splitting of Δ_N in $\mathcal{E}_N$, giving rise to the *Jaynes-Cummings energy ladder* with sets of rungs $\Big\{ |-_N\rangle \,, |+_N\rangle \Big\}$. If the MW field is described by a large amplitude coherent field $|\alpha\rangle = e^{-|\alpha|^2/2} \sum\limits_{n=0}^{\infty} \frac{\alpha^n}{\sqrt{n!}} |n\rangle$, the photon distribution is given by $P(N) = e^{-|\alpha|^2} \frac{\alpha^{2N}}{N!}$, which is centered around $\bar{N} = |\alpha|^2$ with width $\Delta N = \sqrt{\bar{N}}$. The variation of the rung spacing Δ_N does not vary significantly over the spreading of the photon distribution if $\bar{N} \gg 1$. Then, for a large coherent drive strength the dominant splitting can be described by the classical field amplitude, $\Omega_{\text{R}}^{v}\sqrt{\bar{N}} = \Omega_{\text{R}}$, so that $\Delta_N \approx \sqrt{\Omega_{\text{R}}^2 + \delta^2}$ for $N - \bar{N} \ll \Delta N$.
Since the MW dressing does not alter the phonon state, the generic eigenstates at that step are $|\pm_N, M\rangle$.

Before investigating the role of the phonon field, it is important to calculate the effect of the σ_z operator, describing the parametric interaction, on the dressed states. Within one multiplicity $\mathcal{E}(N)$, it can be expressed in the basis $\{|+_N\rangle \,, |-_N\rangle\}$ by:

$$\sigma_z|_{\mathcal{E}(N)} = \begin{pmatrix} \cos^2\theta_N & -\cos\theta_N \sin\theta_N \\ -\cos\theta_N \sin\theta_N & \sin^2\theta_N \end{pmatrix} = \begin{pmatrix} \cos^2\theta_N & -\frac{1}{2}\sin 2\theta_N \\ -\frac{1}{2}\sin 2\theta_N & \sin^2\theta_N \end{pmatrix}. \tag{50}$$

The σ_z operator couples the MW dressed eigenstates: the phonon field described by H_{int} will then be able to induce transitions among the MW dressed states if its frequency Ω_{m} matches the splitting Δ_N. Notice that σ_z does not couple dressed states belonging to different multiplicities ($\langle \pm_N | \, \sigma_z \, |\pm_{N'}\rangle = 0$ if $N \neq N'$).

Dressing the MW dressed states with the phonon field

In the picture of dressed states the role of longitudinal and transversal magnetic field components are interchanged: for near resonant pumping, the dressed spin is polarized along an axis in the equatorial plane of the Bloch sphere. This is a consequence to having rotated the eigenvectors onto the equator of the original Bloch sphere, see equation (49). Hence, when a mechanical oscillation moves the NV in space, the magnetic field experienced by the spin qubit is modulated on timescales on the order of the oscillation period. Both parallel and transverse components of the B field (with respect to the NV quantification axis, z) are modulated by the nanomotion but we only restrict our study to the parametric coupling case. As we will see, our measurement is based on a spectroscopy of the dressed qubit, and the transition frequencies are not affected at first order by perpendicular coupling terms ($\sigma_x(\hat{a}+\hat{a}^\dagger)$). However these terms will have to be taken into account when inspecting the coherence of the dressed qubit (which is beyond the scope of that paper).
Thus the mechanical motion will cause a modulation of the magnetic field along the **z**-direction experienced by the spin oscillating at frequency $\Omega_\mathrm{m}/2\pi$. Thus, it can induce transitions between the dressed states if the field is resonant with their energy splitting $\hbar\Delta_N = \hbar\Omega_\mathrm{R}$. We then lead to develop a second dressing of the dressed qubit with the phonon field.
The hybrid interaction Hamiltonian,

$$H_\mathrm{int} = \hbar g_z(\hat{a}^\dagger + \hat{a})\sigma_z,$$

does not couple multiplicities with different photon number N but permits raising or lowering phonon number M by one unit. Its effect can thus be studied between dressed states combined with adjacent phonon number states ($|\pm_N, M\rangle$). The following table represents the matrix elements of the interaction Hamiltonian (within an overall $\hbar g_z$ factor)

$$\begin{array}{c} |-_N,M+1\rangle \\ |+_N,M\rangle \\ |-_N,M\rangle \\ |+_N,M-1\rangle \\ |-_N,M-1\rangle \\ |+_N,M-2\rangle \end{array}
\overset{\begin{array}{cccccc} |-_N,M+1\rangle & |+_N,M\rangle & |-_N,M\rangle & |+_N,M-1\rangle & |-_N,M-1\rangle & |+_N,M-2\rangle \end{array}}{\begin{pmatrix}
0 & -\frac{\sqrt{M+1}}{2}\sin 2\theta_N & \sqrt{M+1}\sin^2\theta_N & 0 & 0 & 0 \\
-\frac{\sqrt{M+1}}{2}\sin 2\theta_N & 0 & 0 & \sqrt{M}\cos^2\theta_N & -\frac{\sqrt{M}}{2}\sin 2\theta_N & 0 \\
\sqrt{M+1}\sin^2\theta_N & 0 & 0 & -\frac{\sqrt{M}}{2}\sin 2\theta_N & \sqrt{M}\sin^2\theta_N & 0 \\
0 & \sqrt{M}\cos^2\theta_N & -\frac{\sqrt{M}}{2}\sin 2\theta_N & 0 & 0 & \sqrt{M-1}\cos^2\theta_N \\
0 & -\frac{\sqrt{M}}{2}\sin 2\theta_N & \sqrt{M}\sin^2\theta_N & 0 & 0 & -\frac{\sqrt{M-1}}{2}\sin 2\theta_N \\
0 & 0 & 0 & \sqrt{M-1}\cos^2\theta_N & -\frac{\sqrt{M-1}}{2}\sin 2\theta_N & 0
\end{pmatrix}}. \tag{51}$$

Having realized that the action of σ_z can induce state flips in the dressed state basis (contribution of non-diagonal terms in (50), it signifies that the phonon field can now dress the MW dressed basis $|\pm_N\rangle$. It is natural to inspect its effect in term of a dressing of the dressed states with the phonon field and work on the following set of phononic multiplicities:

$$\mathcal{F}(N,M) \equiv \Big\{ |+_N, M-1\rangle\,, |-_N, M\rangle \Big\}. \tag{52}$$

The role of the interaction Hamiltonian is further investigated in Supplementary Figure 3. In addition to the intra-multiplicity coupling terms, it also highlights that the interaction Hamiltonian moreover couples different multiplicities ($\mathcal{F}(N,M) \leftrightarrow \mathcal{F}(N,M\pm1)$).
However, the inter-multiplicity transitions are due to fast rotating coherences and we can neglect their contribution by means of a second rotating wave approximation concerning the phonon field, oscillating at $\Omega_\mathrm{m}/2\pi$. Again such an approximation is valid as long as $g_z\sqrt{M} \ll \Omega_\mathrm{R}$. As explained in Supplementary Figure 3, we are then only left with the intra-multiplicity transitions, $|+_N, M\rangle \Leftrightarrow |-_N, M+1\rangle$, which preserve the global dressed state and phononic excitation number:

Then, the interaction Hamiltonian restricted to each MW dressed multiplicity can be simplified to

$$H_\mathrm{int}^{(\mathrm{N})} = -\frac{\hbar g_z}{2}\frac{\sin 2\Theta_N}{2}\Big(\hat{a}^\dagger |-_N\rangle\langle+_N| + \hat{a}|+_N\rangle\langle-_N|\Big) \tag{53}$$

Its action in the $\mathcal{F}(N,M)$ basis, having uncoupled energies

$$E_{|\pm_N,M\rangle}/\hbar = N\omega - \frac{\delta}{2} \pm \frac{1}{2}\sqrt{\delta^2 + N{\Omega_\mathrm{R}^v}^2} + M\Omega_\mathrm{m}, \tag{54}$$

where an energy offset of $\hbar(N\omega - \delta/2 - \Delta_N/2 + M\Omega_{\rm m})$ has been subtracted, can be written as

$$H^{(N,M)}/\hbar = \begin{pmatrix} \Delta_N - \Omega_{\rm m} & g_{N,M}/2 \\ g_{N,M}/2 & 0 \end{pmatrix}, \tag{55}$$

where $g_{N,M}$ is defined as

$$g_{N,M} \equiv -\frac{g_z\sqrt{M}}{2}\sin 2\Theta_N = -\frac{g_z\sqrt{M}}{2}\sqrt{\frac{N{\Omega^v_{\rm R}}^2}{\delta^2 + N{\Omega^v_{\rm R}}^2}}. \tag{56}$$

The new eigenenergies $E_{|\pm_{N,M}\rangle}$ and eigenstates $|\pm_{N,M}\rangle$ can thus be described by

$$E_{|\pm_{N,M}\rangle} = N\omega - \frac{\delta}{2} + M\Omega_{\rm m} - \frac{\Omega_{\rm m} - \Delta_N}{2} \pm \frac{1}{2}\Delta_{N,M}, \tag{57}$$

where

$$\Delta_{N,M} \equiv \sqrt{(\Omega_{\rm m} - \Delta_N)^2 + g^2_{N,M}} \tag{58}$$

and

$$|+_{N,M}\rangle = \cos\theta_{N,M}\,|+_N, M-1\rangle + \sin\theta_{N,M}\,|-_N, M\rangle\,, \tag{59}$$

$$|-_{N,M}\rangle = -\sin\theta_{N,M}\,|+_N, M-1\rangle + \cos\theta_{N,M}\,|-_N, M\rangle \tag{60}$$

using

$$\tan 2\theta_{N,M} = -\kappa_{N,M}/(\Omega_{\rm m} - \Delta_N) \quad \text{with} \quad \theta_{N,M} \in [0, \pi/2]. \tag{61}$$

For a large MW drive, $\sqrt{N}\Omega^v_{\rm R} \to \Omega_{\rm R}$. Similarly, for large oscillation amplitude $\sqrt{M}g_z \to \delta\omega_0$, $\delta\omega_0$ being the amplitude of the parametric energy modulation caused by the oscillation in the magnetic field gradient. The splitting becomes $\Delta_{N,M} \to \Delta_{\rm Mollow}$, with

$$\Delta_{\rm Mollow} = \sqrt{\left(\Omega_{\rm m} - \sqrt{\delta^2 + \Omega^2_{\rm R}}\right)^2 + \left(\frac{\delta\omega_0}{2}\right)^2 \frac{\Omega^2_{\rm R}}{\delta^2 + \Omega^2_{\rm R}}}. \tag{62}$$

In case of resonant pumping $\delta = 0$, it simplifies to:

$$\Delta_{\rm Mollow} = \sqrt{(\Omega_{\rm m} - \Omega_{\rm R})^2 + \left(\frac{\delta\omega_0}{2}\right)^2}. \tag{63}$$

We note that the mechanical contribution is fully encoded in the $\Omega_{\rm m}, \delta\omega_0$ terms. Its generalization to a mechanical oscillator driven out of resonance can simply be obtained by replacing $\Omega_{\rm m}$ by the classical driving frequency $\Omega_{\rm d}$ and $\delta\omega_0$ by the corresponding parametric coupling strength $\delta\omega_0[\Omega_{\rm d}]$ whose derivation has been explained in the main text and which depends on the spin qubit trajectory in space. Also the extension to a driven multimode resonator is described in the main text.

Frequencies of Rabi oscillations

The frequencies present in the Rabi oscillations correspond to the energy differences between the doubly dressed states of neighbouring multiplicities coupled by the σ_z operator.

$$\begin{cases} |+_{N,M+1}\rangle \leftrightarrow |-_{N,M}\rangle \text{ oscillating at } \Omega_{\rm m} + \Delta_{N,M} \\ |-_{N,M+1}\rangle \leftrightarrow |-_{N,M}\rangle \text{ oscillating at } \Omega_{\rm m} \\ |+_{N,M+1}\rangle \leftrightarrow |+_{N,M}\rangle \text{ oscillating at } \Omega_{\rm m} \\ |-_{N,M+1}\rangle \leftrightarrow |+_{N,M}\rangle \text{ oscillating at } \Omega_{\rm m} - \Delta_{N,M} \\ |+_{N,M}\rangle \leftrightarrow |-_{N,M}\rangle \text{ oscillating at } \Delta_{N,M} \end{cases} \tag{64}$$

The frequencies indicated above indeed well reproduce the observed components in the Fourier spectra of Rabi oscillations. We point out that the observation of the intramultiplicity transition at frequency $\Delta_{N,M}$ is only possible when the MW detuning is non-zero ($\delta \neq 0$). Dependency on $\Omega_{\rm R}$ and $\delta\omega_0$ is verified in Supplementary Figure 4.

Interpretation of Rabi oscillation spectra in terms of dressed spin fluorescence

In this section we further analyze the experimental observations. It is important to recall that at resonance ($\delta = 0$) the MW dressed qubit is aligned along the **x**-axis of the Bloch sphere representing the original spin qubit. As we have explained, the roles of the σ_x and σ_z operator for the dressed spin are interchanged and the mechanical motion along the **z**-axis resonantly couples to the dressed states. The MW dressed qubit therefore experiences irradiation from the coherent phonon field oscillating at $\Omega_{\rm m}$. Here, we describe the evolution of the dressed spin by a quantum master equation. We introduce Bloch equations for the MW dressed qubit coupled to the phonon field. This permits justifying the above mentionned rotation of the point of view in the Bloch sphere operated by the MW dressing. This also allows analyzing the spin locking signatures: in particular, we here find the dependence of the weights of the Mollow triplet peaks on the oscillation amplitude and phase.
Finally, this theoretical derivation permits to justify that the measurement of the Rabi oscillation of the bare qubit permits to measure the temporal evolution of the dipole of the MW dressed qubit. Fourier Transforms of such traces thus correspond precisely to the scenario of observing a Mollow triplet: when the fluorescence of a two level system is measured in presence of an intense pumping field which resonantly drives the TLS.

The reduced density matrix for the dressed qubit

Experimentally, we measure the temporal evolution of σ_z:

$$\begin{aligned} \langle \sigma_z(t) \rangle &= \mathrm{Tr}\left\{ \rho \sigma_z \right\} \\ &= \sum_{\substack{\alpha=\pm \\ \beta=\pm \\ N \\ N'}} \langle \alpha_N | \, \rho \, | \beta_{N'} \rangle \underbrace{\langle \beta_{N'} | \, \sigma_z \, | \alpha_N \rangle}_{\text{A}} . \end{aligned} \tag{65}$$

Considering an example for A:

$$\begin{aligned} A = \langle +_{N'} | \, \sigma_z \, | -_N \rangle &= \left(\cos\theta_{N'} \langle 1, N'-1 | + \sin\theta_{N'} \langle 0, N' | \right) \sigma_z \left(-\sin\theta_{N'} | 1, N'-1 \rangle + \cos\theta_{N'} | 0, N' \rangle \right) \\ &= -\cos\theta_{N'} \sin\theta_N \langle 1 | \, \sigma_z \, | 1 \rangle \, \delta_{N,N'} \\ &= -\cos\theta_N \sin_N \delta_{N,N'} , \end{aligned}$$

we see that all parts of the sum with $N \neq M$ vanish. Thus,

$$\langle \sigma_z(t) \rangle = \sum_{\substack{\alpha=\pm \\ \beta=\pm \\ N}} \langle \alpha_N | \, \rho \, | \beta_N \rangle \langle \beta_N | \, \sigma_z \, | \alpha_N \rangle = \sum_N \mathrm{Tr} \left\{ \rho^{(N)} \sigma_z |_{\mathcal{E}(N)} \right\} ,$$

where $\rho^{(N)}$ is the density matrix reduced to the $N^{\rm th}$ multiplicity,

$$\rho^{(N)} = \begin{pmatrix} \rho^N_{++} & \rho^N_{+-} \\ \rho^N_{-+} & \rho^N_{--} \end{pmatrix} . \tag{66}$$

with $\rho^N_{++} = \langle +_N | \, \rho \, | +_N \rangle$. This leads to:

$$\langle \sigma_z(t) \rangle = \sum_N \cos^2\theta_N \rho^{(N)}_{--} + \sin^2\theta_N \rho^{(N)}_{++} - \cos\theta_N \sin\theta_N \left(\rho^{(N)}_{+-} + \rho^{(N)}_{-+} \right) , \tag{67}$$

which reduces in case of resonant pumping to:

$$\langle\sigma_z(t)\rangle = \frac{1}{2}\sum_N(\rho_{--}^{(N)}+\rho_{++}^{(N)}) - \frac{1}{2}\sum_N(\rho_{+-}^{(N)}+\rho_{-+}^{(N)})$$

$$= \frac{1}{2}(\pi_- + \pi_+) - \frac{1}{2}(\sigma_{+-}+\sigma_{-+}) \tag{68}$$

$$= \frac{1}{2} - \frac{1}{2}(\sigma_{+-}+\sigma_{-+}). \tag{69}$$

Here we have defined the reduced populations and coherences:

$$\pi_\pm \equiv \sum_N \rho_{\pm\pm}^{(N)} \qquad \sigma_{+-} \equiv \sum_N \rho_{+-}^{(N)},$$

which illustrates that we can introduce an effective two level system, which entirely describes the MW dressed qubit in case of large MW strength. The full derivation is given in [6]. Equation (68) signifies that **Rabi oscillations can be understood as a measurement of the temporal evolution of the dipole or coherence** ($\propto \sigma_{+-}+\sigma_{-+}$) **of the MW dressed qubit**. The spectrum of the Rabi oscillation thus represents the equivalent of a fluorescence spectrum of the MW dressed qubit. Note that in contrast to the original Mollow experiment, the Mollow triplet is not observed in a measurement of the side fluorescence but directly in the time resolved evolution of the dressed qubit dipole. We can therefore expect to be able to interpret spectra of Rabi oscillations in terms of phenomena similar to spectra of photons emitted by a single atom irradiated by a light field.

Master equation for the dressed qubit

The dressed states $\{|\pm_N\rangle\}$ forms a base of the spin MW subsystem. We describe here the dynamics of the pseudo qubit in terms of Bloch equations in presence of a parametric modulation originating from mechanical motion. The global density matrix ρ follows the evolution equation

$$\dot\rho = \frac{1}{i\hbar}[H_0 + H_{\rm int}, \rho] + \dot\rho_{\rm relax} \tag{70}$$

with

$$H_0 + H_{\rm int} = \sum_N \Big(|+_N\rangle\langle +_N| E_{+_N} + |-_N\rangle\langle -_N| E_{-_N}\Big) + \hbar g_z \left(a^\dagger + a\right)\sigma_z. \tag{71}$$

If a large enough MW field is applied ($\bar N \gg \Delta N \gg 1$), which is the case in most experimental conditions when operating with a NV spin qubit, the variation of θ_N over the dominantly populated multiplicities is negligible. Following the approximation explained previously, we can restrict our analysis to one single multiplicity with $N \approx \bar N$.

For a coherent mechanical oscillation, generating a coherent parametric modulation strength $\delta\omega_0$, we can rewrite $\hbar g_z\left(a^\dagger + a\right)\sigma_z$ as $\hbar\delta\omega_0\cos(\Omega_{\rm m}t+\alpha)\sigma_z$ (where α is now the initial phase of the RF field). In that situation, we have

$$\begin{aligned}\dot\rho_{+-}^N &\equiv \langle +_N|\,\dot\rho\,|-_N\rangle \\ &= \frac{1}{i\hbar}\langle +_N|\,[H_0,\rho]\,|-_N\rangle - i\delta\omega_0\cos\left(\Omega_{\rm m}t+\alpha\right)\langle +_N|\,\sigma_z\rho - \rho\sigma_z\,|-_N\rangle + \dot\rho^N_{+-,\rm relax} \\ &= -i\frac{E_{+_N}-E_{-_N}}{\hbar}\rho_{+-}^N + i\frac{\delta\omega_0}{2}\cos(\Omega_{\rm m}t+\alpha)\left(\rho_{--}^N - \rho_{++}^N\right) + \dot\rho^N_{+-,\rm relax}.\end{aligned}$$

Bloch equations describing the dressed qubit evolution

The damping mechanisms acting on the dressed qubit are not the same as the one involved in the original qubit, see for example [7]. The changes in the dressed qubit frequency and its orientation on the Bloch sphere make the dressed qubit sensitive to environmental noises at different frequencies and along different orientations. Here, we will introduce the decoherence rate $\Gamma_{\rm spin}$ of the dressed qubit and and simplify the dressed state energy decay rate to

$2\Gamma_{\rm spin}$, which is assuming that T_1 processes of the dressed spin qubit are negligible compared to T_2 processes. We thus arrive at a set of Bloch equations for the dressed states

$$\begin{aligned}
\dot{\rho}^N_{+-} &= -i\Omega_{\rm R}\rho^N_{+-} - i\frac{\delta\omega_0}{2}\cos(\Omega_{\rm m}t+\alpha)\left(\rho^N_{++}-\rho^N_{--}\right) - \Gamma_{\rm spin}\rho^N_{+-},\\
\dot{\rho}^N_{-+} &= +i\Omega_{\rm R}\rho^N_{+-} + i\frac{\delta\omega_0}{2}\cos(\Omega_{\rm m}t+\alpha)\left(\rho^N_{++}-\rho^N_{--}\right) - \Gamma_{\rm spin}\rho^N_{-+},\\
\dot{\rho}^N_{++} &= i\frac{\delta\omega_0}{2}\cos(\Omega_{\rm m}t+\alpha)\left(\rho^N_{-+}-\rho^N_{+-}\right) - 2\Gamma_{\rm spin}\rho^N_{++},
\end{aligned} \tag{72}$$

which can be summed over N in order to yield the Bloch equations of the pseudo spin:

$$\begin{aligned}
\dot{\sigma}_{+-} &= -i\Omega_{\rm R}\sigma_{+-} - i\frac{\delta\omega_0}{2}\cos(\Omega_{\rm m}t+\alpha)\left(\pi_{++}-\pi_{--}\right) - \Gamma_{\rm spin}\sigma_{+-},\\
\dot{\sigma}_{-+} &= +i\Omega_{\rm R}\sigma_{-+} + i\frac{\delta\omega_0}{2}\cos(\Omega_{\rm m}t+\alpha)\left(\pi_{++}-\pi_{--}\right) - \Gamma_{\rm spin}\sigma_{-+},\\
\dot{\pi}_{++} &= i\frac{\delta\omega_0}{2}\cos(\Omega_{\rm m}t+\alpha)\left(\sigma_{-+}-\sigma_{+-}\right) - 2\Gamma_{\rm spin}\pi_{++}.
\end{aligned} \tag{73}$$

These equations are commonly solved by means of a rotating wave approximation. In that case we express (73) in terms of the coherences in the frame rotating at the mechanical oscillator frequency, defining

$$\tilde{\sigma}^\alpha_{-+} = \sigma_{-+}e^{-i\Omega_{\rm m}t-i\alpha},$$

for which the Bloch equations in the rotating frame are

$$\begin{aligned}
\dot{\tilde{\sigma}}^\alpha_{+-} &= -i(\Omega_{\rm R}-\Omega_{\rm m})\tilde{\sigma}^\alpha_{+-} - i\frac{\delta\omega_0}{4}\left(\pi_{++}-\pi_{--}\right) - \Gamma_{\rm spin}\tilde{\sigma}^\alpha_{+-},\\
\dot{\tilde{\sigma}}^\alpha_{-+} &= +i(\Omega_{\rm R}-\Omega_{\rm m})\tilde{\sigma}^\alpha_{-+} + i\frac{\delta\omega_0}{4}\left(\pi_{++}-\pi_{--}\right) - \Gamma_{\rm spin}\tilde{\sigma}^\alpha_{-+},\\
\dot{\pi}_{++} &= i\frac{\delta\omega_0}{4}\left(\tilde{\sigma}^\alpha_{-+}-\tilde{\sigma}^\alpha_{+-}\right) - 2\Gamma_{\rm spin}\pi_{++}.
\end{aligned} \tag{74}$$

We then define the Bloch vector $\mathbf{b}_\alpha$ as:

$$\mathbf{b}_\alpha = \begin{pmatrix} u_\alpha \\ v_\alpha \\ w_\alpha \end{pmatrix} = \begin{pmatrix} (\tilde{\sigma}^\alpha_{-+}+\tilde{\sigma}^\alpha_{+-})/2 \\ (\tilde{\sigma}^\alpha_{-+}-\tilde{\sigma}^\alpha_{+-})/2i \\ (\pi_{++}-\pi_{--})/2 \end{pmatrix}, \tag{75}$$

so that

$$\begin{aligned}
\sigma^\alpha_{-+} &= e^{i\Omega_{\rm m}t+i\alpha}(u_\alpha+iv_\alpha),\\
\sigma^\alpha_{+-} &= e^{-i\Omega_{\rm m}t-i\alpha}(u_\alpha-iv_\alpha),
\end{aligned}$$

and

$$\frac{\sigma^\alpha_{+-}(t)+\sigma^\alpha_{-+}(t)}{2} = u_\alpha(t)\cos(\Omega_{\rm m}t+\alpha) - v_\alpha(t)\sin(\Omega_{\rm m}t+\alpha),$$

which is the quantity measured through the Rabi oscillations of the original qubit (see equation (68)). The equation of evolution of the Bloch vector is then

$$\begin{pmatrix} \dot{u}_\alpha \\ \dot{v}_\alpha \\ \dot{w}_\alpha \end{pmatrix} = \begin{pmatrix} -\Gamma_{\rm spin} & (\Omega_{\rm m}-\Omega_{\rm R}) & 0 \\ -(\Omega_{\rm m}-\Omega_{\rm R}) & -\Gamma_{\rm spin} & -\delta\omega_0/2 \\ 0 & \delta\omega_0/2 & -2\Gamma_{\rm spin} \end{pmatrix} \begin{pmatrix} u_\alpha \\ v_\alpha \\ w_\alpha \end{pmatrix}. \tag{76}$$

Notice that the dependence on the initial phase α is hidden in the initial conditions at $t=0$. Since in our protocol, the spin is always prepared in the ground state $|0\rangle$ by optical pumping, we have

$$\rho^{(N)}(t=0) = \frac{1}{2}\begin{pmatrix} 1 & 1 \\ 1 & 1 \end{pmatrix}, \tag{77}$$

and

$$u_\alpha(t=0) = \frac{1}{2}\left(\frac{e^{-i\alpha}}{2} + \frac{e^{i\alpha}}{2}\right) = \frac{\cos\alpha}{2},$$
$$v_\alpha(t=0) = \frac{1}{2i}\left(\frac{e^{-i\alpha}}{2} - \frac{e^{i\alpha}}{2}\right) = -\frac{\sin\alpha}{2},$$
$$w_\alpha(t=0) = 0.$$

We will restrict the following considerations to the resonant condition, where $\Omega_\mathrm{m} = \Omega_\mathrm{R}$. We then look for solutions like

$$\begin{pmatrix} u_\alpha \\ v_\alpha \\ w_\alpha \end{pmatrix}(t) \propto e^{\lambda t}$$

and find solutions for λ by solving the eigenvalue problem $\det(M - \lambda\mathbb{I}) = 0$. The eigenvalues are

$$\lambda_0 = -\Gamma_\mathrm{spin}, \tag{78}$$
$$\lambda_\pm = -\frac{3}{2}\Gamma_\mathrm{spin} \pm \frac{1}{2}\sqrt{\Gamma_\mathrm{spin}^2 - \delta\omega_0^2}, \tag{79}$$

for which the Bloch vector evolves as

$$u_\alpha(t) = \frac{\cos\alpha}{2} e^{-\Gamma_\mathrm{spin} t}, \tag{80}$$
$$v_\alpha(t) = -\frac{\sin\alpha}{2} e^{-\frac{3}{2}\Gamma_\mathrm{spin} t}\left(\cosh\left(\frac{1}{2}\sqrt{\Gamma_\mathrm{spin}^2 - \delta\omega_0^2 t}\right) + \frac{\Gamma_\mathrm{spin}}{\sqrt{\Gamma_\mathrm{spin}^2 - \delta\omega_0^2}}\sinh\left(\frac{1}{2}\sqrt{\Gamma_\mathrm{spin}^2 - \delta\omega_0^2 t}\right)\right), \tag{81}$$
$$w_\alpha(t) = \frac{\delta\omega_0}{2}\sin\alpha \frac{e^{-\frac{3}{2}\Gamma_\mathrm{spin} t}}{\sqrt{\Gamma_\mathrm{spin}^2 - \delta\omega_0^2}}\sinh\left(\frac{1}{2}\sqrt{\Gamma_\mathrm{spin}^2 - \delta\omega_0^2 t}\right). \tag{82}$$

We note that for large enough amplitudes $\delta\omega_0$, the character of the evolution changes from an exponential decay to exponentially decaying oscillations. When $\delta\omega_0 > \Gamma_\mathrm{spin}$,

$$\cosh\left(\frac{1}{2}\sqrt{\Gamma_\mathrm{spin}^2 - \delta\omega_0^2 t}\right) \to \cos\left(\frac{1}{2}\sqrt{\delta\omega_0^2 - \Gamma_\mathrm{spin}^2 t}\right),$$
$$\frac{1}{\sqrt{\Gamma_\mathrm{spin}^2 - \delta\omega_0^2}}\sinh\left(\frac{1}{2}\sqrt{\Gamma_\mathrm{spin}^2 - \delta\omega_0^2 t}\right) \to \frac{1}{\sqrt{\delta\omega_0^2 - \Gamma_\mathrm{spin}^2}}\sin\left(\frac{1}{2}\sqrt{\delta\omega_0^2 - \Gamma_\mathrm{spin}^2 t}\right).$$

The measured Rabi oscillation can be described by:

$$\begin{aligned}\langle\sigma_z^\alpha\rangle(t) &= \sum_N \left(\frac{1}{2} - \frac{\rho_{+-}^N(t) + \rho_{-+}^N(t)}{2}\right) \\ &= \frac{1}{2} - \big(\cos(\Omega_\mathrm{m} t + \alpha)\, u_\alpha(t) - \sin(\Omega_\mathrm{m} t + \alpha)\, v_\alpha(t)\big),\end{aligned} \tag{83}$$

where it is clear that u_α is the component oscillating in phase with the mechanical motion and v_α in quadrature:

$$\begin{aligned}\langle\sigma_z^\alpha\rangle(t) = \frac{1}{2}\Bigg\{1 &- e^{-\Gamma_\mathrm{spin} t}\cos\alpha\cos(\Omega_\mathrm{m} t + \alpha) \\ &- e^{-\frac{3}{2}\Gamma_\mathrm{spin} t}\sin\alpha\sin(\Omega_\mathrm{m} t + \alpha)\Bigg[\cosh\left(\frac{1}{2}\sqrt{\Gamma_\mathrm{spin}^2 - \delta\omega_0^2 t}\right) \\ &+ \frac{\Gamma_\mathrm{spin}}{\sqrt{\Gamma_\mathrm{spin}^2 - \delta\omega_0^2}}\sinh\left(\frac{1}{2}\sqrt{\Gamma_\mathrm{spin}^2 - \delta\omega_0^2 t}\right)\Bigg]\Bigg\},\end{aligned} \tag{84}$$

which we can reexpress as:

$$\langle \sigma_z^\alpha \rangle (t) = \frac{1}{2}\Bigg\{ 1 - e^{-\Gamma_{\text{spin}} t} \cos\alpha \cos\left(\Omega_{\text{m}} t + \alpha\right) - \frac{1}{2} e^{-\frac{3}{2}\Gamma_{\text{spin}} t} \sin\alpha \sin\left(\Omega_{\text{m}} t + \alpha\right) \left[e^{\frac{1}{2}\sqrt{\Gamma_{\text{spin}}^2 - \delta\omega_0^2}\, t} \left(1 + \frac{\Gamma_{\text{spin}}}{\sqrt{\Gamma_{\text{spin}}^2 - \delta\omega_0^2}} \right) + e^{-\frac{1}{2}\sqrt{\Gamma_{\text{spin}}^2 - \delta\omega_0^2}\, t} \left(1 - \frac{\Gamma_{\text{spin}}}{\sqrt{\Gamma_{\text{spin}}^2 - \delta\omega_0^2}} \right) \right] \Bigg\}. \tag{85}$$

With our definition of the σ_z operator, the population $P_0^\alpha = 1 - \langle \sigma_z^\alpha \rangle (t)$ is evolving as

$$P_0^\alpha(t) = \frac{1}{2}\Bigg\{ 1 + e^{-\Gamma_{\text{spin}} t} \cos\alpha \cos\left(\Omega_{\text{m}} t + \alpha\right) + \frac{1}{2} e^{-\frac{3}{2}\Gamma_{\text{spin}} t} \sin\alpha \sin\left(\Omega_{\text{m}} t + \alpha\right) \left[e^{\frac{1}{2}\sqrt{\Gamma_{\text{spin}}^2 - \delta\omega_0^2}\, t} \left(1 + \frac{\Gamma_{\text{spin}}}{\sqrt{\Gamma_{\text{spin}}^2 - \delta\omega_0^2}} \right) + e^{-\frac{1}{2}\sqrt{\Gamma_{\text{spin}}^2 - \delta\omega_0^2}\, t} \left(1 - \frac{\Gamma_{\text{spin}}}{\sqrt{\Gamma_{\text{spin}}^2 - \delta\omega_0^2}} \right) \right] \Bigg\}. \tag{86}$$

Comparison with the experimental observations

Experimentally, our measurement protocols are not synchronized to the oscillatory phase, so that we have to average over α in order to recover the observed spin dynamics:

$$P_0(t) = \frac{1}{2\pi} \int_0^{2\pi} \mathrm{d}\alpha\, P_0^\alpha(t). \tag{87}$$

Using

$$\frac{1}{2\pi} \int_0^{2\pi} \mathrm{d}\alpha\, \cos\left(\Omega_{\text{m}} t + \alpha\right) \cos\alpha = \frac{\cos\Omega_{\text{m}} t}{2} \quad \text{and} \quad \frac{1}{2\pi} \int_0^{2\pi} \mathrm{d}\alpha \sin\left(\Omega_{\text{m}} t + \alpha\right) \sin\alpha = \frac{\cos\Omega_{\text{m}} t}{2},$$

we get

$$P_0(t) = \frac{1}{2} + \frac{1}{4} e^{-\Gamma_{\text{spin}} t} \cos\Omega_{\text{m}} t + \frac{1}{8} e^{-\frac{3}{2}\Gamma_{\text{spin}} t} \cos\Omega_{\text{m}} t \left[e^{\frac{1}{2}\sqrt{\Gamma_{\text{spin}}^2 - \delta\omega_0^2}\, t} \left(1 + \frac{\Gamma_{\text{spin}}}{\sqrt{\Gamma_{\text{spin}}^2 - \delta\omega_0^2}} \right) + e^{-\frac{1}{2}\sqrt{\Gamma_{\text{spin}}^2 - \delta\omega_0^2}\, t} \left(1 - \frac{\Gamma_{\text{spin}}}{\sqrt{\Gamma_{\text{spin}}^2 - \delta\omega_0^2}} \right) \right]. \tag{88}$$

For sufficiently large amplitudes, $\delta\omega_0 > \Gamma_{\rm spin}$, when the Mollow triplet appears, the expression simplifies to

$$\begin{aligned} P_0(t) = \frac{1}{2} + \frac{1}{4}e^{-\Gamma_{\rm spin}t}\cos\left(\Omega_{\rm m}t\right) \\ + \frac{1}{8}e^{-\frac{3}{2}\Gamma_{\rm spin}t}\cos\left(\left(\Omega_{\rm m} + \frac{\delta\omega_0}{2}\right)t\right) \\ + \frac{1}{8}e^{-\frac{3}{2}\Gamma_{\rm spin}t}\cos\left(\left(\Omega_{\rm m} - \frac{\delta\omega_0}{2}\right)t\right) \end{aligned} \quad (89)$$

The three peaks of the Mollow triplet are present in the above equation, as well as their weights, widths and relative phases (which is preserved despite averaging over the mechanical phase α). Notice that the constant term reflects the mean value of Rabi oscillations: $P_0(t \to \infty) = P_1(t \to \infty) = \frac{1}{2}$. By transforming expression (89) into its Fourier space we can write down the analytic form of the *phonon Mollow triplet*:

$$2\pi P_0[\Omega] = \frac{1}{2}\delta[\Omega] + \frac{\frac{1}{8}}{\frac{3}{2}\Gamma_{\rm spin} + i(\Omega - \Omega_{\rm m} + \frac{\delta\omega_0}{2})} + \frac{\frac{1}{4}}{\Gamma_{\rm spin} + i(\Omega - \Omega_{\rm m})} + \frac{\frac{1}{8}}{\frac{3}{2}\Gamma_{\rm spin} + i(\Omega - \Omega_{\rm m} - \frac{\delta\omega_0}{2})}. \quad (90)$$

Equation (90) fully captures amplitude, frequency and relative phase of each component of the Mollow triplet at the spin locking condition. We note that the frequencies of each peak are in agreement with the expressions obtained from the double dressing approach. Expression (90) was used to fit the experimentally measured phononic Mollow triplets with adjustable weights.

Weak and strong coupling of the parametric interaction

Expression (90) describes Rabi spectra taken at the resonance or *spin locking condition* $\Omega_{\rm m} - \Omega_{\rm R} = 0$. This corresponds to the experiment shown in Figure 3 of the article in which the oscillation strength $\delta\omega_0$ is gradually increased. The emergence of the triplet structure with augmenting oscillation amplitude is visible. Since the separation of the sidebands is directly related to the coupling strength, we expect that the individual components are distinguishable once the parametric modulation strength is larger than the peak linewidth: $\delta\omega_0 \gtrsim \Gamma_{\rm spin}$. Such a condition is usually called a strong coupling condition, which applies here to the resonant interaction of the phonon field with respect to the MW dressed spin. It is possible to obtain a spatial interpretation of this criterion: it suggests that the oscillation amplitude projected along the parametric coupling vector $\mathbf{e}_\lambda$ is larger than the Mollow length ($\delta r_{\rm Mollow} \equiv \Gamma_{\rm spin}/|\boldsymbol{\nabla}\omega_0|$, see text):

$$\delta\mathbf{r}\cdot\mathbf{e}_\lambda > \delta r_{\rm Mollow} \quad (91)$$

Sideband asymmetry

The measured Mollow triplets present a systematic asymmetry in the sideband heights, which is well reproduced by numerical simulations (see Figure 4d and Supplementary Figure 4). This asymmetry is absent in the numerical simulations when the rotating wave approximation is realized, as can be seen in the analytical expressions exposed above. It is a consequence of the Bloch-Siegert mechanism, a "phononic light shift" due fast counter rotating terms in the dressed qubit-phonon interaction, which renormalizes the pseudo qubit energy splitting $\Omega_{\rm R}$ when it is strongly driven by the phonon field [8, 9].

In the experiment the observed height asymmetry may also occur due to other experimental imperfections. For example, the relative sideband amplitude depends strongly on the detuning ($\Omega_{\rm d} - \Omega_{\rm d}$) between the mechanical drive and Rabi frequencies as evidenced in the article figure 3d and in the σ_z matrix elements in the doubly dressed base. In the experiment presented by figure 4a, the Rabi frequency has been adjusted each time to the mechanical drive frequency by tuning the microwave power. Any experimental errors originating from the calibration of the power dependence of the Rabi frequency could lead to a small but finite and uncontrolled detuning between Ω_R and $\Omega_{\rm d}$ which could cause a sideband height asymmetry. However the observed sideband amplitude ratio can be as large as 50%, which cannot be fully explained by this simple artefact since it would require a too large detuning.

Indeed our experimental tuning procedure doesn't take into account the Bloch-Siegert shift of the dressed spin qubit frequency, as observed in [8] with a RF driving field. The Bloch-Siegert shift starts to play a role as soon as the coupling strength becomes comparable to the spin energy splitting. In our system, coupling strengths on the order

of the dressed qubit energy ($\delta\omega_0 \approx \Omega_{\rm R}$) are experimentally obtained. It can be shown, see [10] that the maximum in the magnitude of the central peak is displaced towards larger microwave powers by $\Omega_{\rm R}^{\rm BS} = \Omega_{\rm m} + \frac{\delta\omega_0^2}{16\Omega_{\rm m}}$ which can lead to a detuning as large as 750 kHz for a coupling strength of 8 MHz. As a consequence, this creates a sideband assymetry that increases with the coupling strength and can reach up to 50% in our case. When adding an inherent experimental noise floor to these theoretical spectra, the upper sideband will be vanishing at large separation, i.e. around the upper mechanical resonance.
It is important to mention that the correction due to Bloch-Siegert effects on the measured parametric modulation strength $\delta\omega_0$ occurs at the second order in $\delta\omega_0/\Omega_{\rm m}$ since we lie (see Figure 3d) in the parabolic section of the Mollow sidebands (numerical simulations put an upper bound of 8% on the relative error made). As a consequence, it is justified to neglect the Bloch-Siegert shift on the estimation of $\delta\omega_0$, even if it can play an important role on the sidebands amplitude asymmetry. In the experiment, we have chosen the experimental procedure consisting in tuning the Rabi frequency to the mechanical drive frequency at each step, without compensating from the potential Bloch-Siegert shift. This experiment is aiming at measuring experimentally the coupling strength $\delta\omega_0$ for each mechanical drive frequency. In that sense the inherent Bloch-Siegert shift of the Rabi frequency cannot be anticipated in advance without knowing the actual value of $\delta\omega_0$.

Supplementary References

[1] A. Gloppe, P. Verlot, E. Dupont-Ferrier, A. Siria, P. Poncharal, G. Bachelier, P. Vincent and O. Arcizet, Bidimensional nano-optomechanics and topological backaction in a non-conservative radiation force field, Nature Nano. **9**, 920-926 (2014).

[2] O. Arcizet, V. Jacques, A. Siria, P. Poncharal, P. Vincent, and S. Seidelin, A single nitrogen-vacancy defect coupled to a nanomechanical oscillator, Nature Phys. **7**, 879-883 (2011).

[3] J.-P. Tetienne, L. Rondin, P. Spinicelli, M. Chipaux, T. Debuisschert, J.-F. Roch and V. Jacques, Magnetic-field-dependent photodynamics of single NV defects in diamond : an application to qualitative all-optical magnetic imaging, New Journal of Physics **14**, 103033 (2012).

[4] A. Dreau, M. Lesik, L. Rondin, P. Spinicelli, O. Arcizet, J.-F. Roch and V. Jacques, Avoiding power broadening in optically detected magnetic resonance of single NV defects for enhanced DC magnetic field sensitivity, Physical Review B **84**, 195204 (2011).

[5] S. Haroche and J. M. Raimond, Exploring the quantum (Oxford University Press, 2006).

[6] C. Cohen-Tannoudji, J. Dupont-Roc, and G. Grynberg, Photons and Atoms : Introduction to Quantum Electrodynamics (Wiley, 2004).

[7] G. Ithier, E. Collin, P. Joyez, P. Meeson, D. Vion, D. Esteve, F. Chiarello, A. Shnirman, Y. Makhlin, J. Schrifl , and G. Schön, Decoherence in a superconducting quantum bit circuit, Physical Review B **72**, 134519 (2005).

[8] S. Rohr, E. Dupont-Ferrier, B. Pigeau, P. Verlot, V. Jacques, and O. Arcizet, Synchronizing the dynamics of a single nitrogen vacancy spin qubit on a parametrically coupled radio-frequency field through microwave dressing, Phys. Rev. Lett. **112**, 010502 (2014).

[9] A. Saiko and G. Fedoruk, Effect of the Bloch-Siegert shift on the frequency responses of Rabi oscillations in the case of nutation resonance, JETP Letters **3**, 128 (2008).

[10] C. Cohen-Tannoudji et al., Quantum calculation of the higher order terms in the Bloch-Siegert shift," J. Phys. B : Atom. Molec. Phys. **6**, 214-217 (1973).
